# Identification of DNA-Methylated CpG Islands Associated With Gene Silencing in the Adult Body Tissues of the Ogye Chicken Using RNA-Seq and Reduced Representation Bisulfite Sequencing

**DOI:** 10.3389/fgene.2019.00346

**Published:** 2019-04-16

**Authors:** Won-Jun Lim, Kyoung Hyoun Kim, Jae-Yoon Kim, Seongmun Jeong, Namshin Kim

**Affiliations:** ^1^Genome Editing Research Center, Korea Research Institute of Bioscience and Biotechnology, Daejeon, South Korea; ^2^Department of Bioinformatics, KRIBB School of Bioscience, University of Science and Technology, Daejeon, South Korea

**Keywords:** gene silencing, DNA methylation, RNA-seq, RRBS – reduced representation bisulfite sequencing, transcriptome, methylome

## Abstract

DNA methylation is an epigenetic mark that plays an essential role in regulating gene expression. CpG islands are DNA methylations regions in promoters known to regulate gene expression through transcriptional silencing of the corresponding gene. DNA methylation at CpG islands is crucial for gene expression and tissue-specific processes. At the current time, a limited number of studies have reported on gene expression associated with DNA methylation in diverse adult tissues at the genome-wide level. Expression levels are rarely affected by DNA methylation in normal adult tissues; however, statistical differences in gene expression level correlated with DNA methylation have recently been revealed. In this study, we examined 20 pairs of DNA methylomes and transcriptomes from RNA-seq and reduced representation bisulfite sequencing (RRBS) data using adult Ogye chicken tissues. A total of 3,133 CpG islands were identified from 20 tissue data in a single chicken sample which could affect downstream genes. Analyzing these CpG island and gene pairs, 121 significant units were statistically correlated. Among them, six genes (*CLDN3, DECR2, EVA1B, NME4, NTSR1*, and *XPNPEP2*) were highly significantly changed by altered DNA methylation. Finally, our data demonstrated how DNA methylation correlated to gene expression in normal adult tissues. Our source codes can be found at https://github.com/wjlim/correlation-between-rna-seq-and-RRBS.

## Introduction

The development of next generation sequencing (NGS) has allowed the investigation of genomes of a large number of species at high quality and resolution. Genomic researches along with transcriptome and DNA methylome has enabled us to deepen our knowledge of functional studies at work in complex genomics (Kulski, 2016). Epigenomic regulation occurs across species, and animal models can be used to infer potential human disease related epigenetic regulation (Robertson, 2005). In particular, the chicken model is a practical model system widely used for vertebrate research topics (Li et al., 2011; Hu et al., 2013). Although the genomic and transcriptomic landscapes of livestock animals such as cows, goats, pigs, and chickens have been constructed (Kern et al., 2018), genome wide level in diverse tissue specific DNA methylation studies remains unknown.

DNA methylation constitutes a major epigenetic modification of the genome and is essential for cellular reprogramming, tissue differentiation, and normal development related to many biological processes including gene expression regulation. DNA methylation is known to occur at the 5′ of cytosine in CpG dinucleotides which are found mostly in so called CGI regions present in promoters. The DNA methylation of CGIs regulates gene expression by transcriptional silencing of the corresponding gene (Newell-Price et al., 2000; Curradi et al., 2002; Ehrlich and Lacey, 2013). DNA methylation is also crucial for gene expression and tissue-specific processes (Wilkinson, 2015). Some studies have reported that the alteration of DNA methylation affects embryonic development, genomic imprinting, genome stability, and chromatin status (Robertson, 2005). Moreover, epigenetic mutations have been studied in many human diseases. Additionally, studies have revealed changes in the DNA methylation profile in many types of tumors (Esteller and Herman, 2002; Sharma et al., 2008; De Jager et al., 2014).

Discovering DNA methylation using RRBS is an effective high-throughput technique for detecting the status of DNA methylation in CGIs. Unlike WGBS, restriction enzymes are used to extract high CpG contents regions, representing only ∼1% of the total DNA methylome (Meissner et al., 2005). However, these sequences cover about 30% of all CpG sites, and these CpG sites account for ∼65% of the promoter CpGs of the entire gene, thus allowing limited but effective genome comparison at a lower cost than WGBS (Gu et al., 2011). CGIs present in the promoter can regulate downstream genes; however, only a few such genes are known to function, at the genome-wide level. While RNA-seq and RRBS can both be used for quantitative analysis, when comparing different adult tissues, there are as many as ∼8,000 statistically DEGs, but there are only ∼100 DMSs in the promoter region (Bird, 2002). A previous study demonstrated that DNA methylation level and gene expression are negatively correlated (Wagner et al., 2014). However, this relationship is rarely known diverse tissues at genome-wide level.

In this study, we examined the Ogye chicken, a domesticated chicken with black skin, fascia, and cockscomb, in Korea. We computed a transcriptome and DNA methylome quantitative matrix using two different types of sequencing platforms—-RNA-seq and RRBS. RNA-Seq facilitates the quantification of the entire transcriptome even in a genome that does not possess a reference sequence. It also provides information on isoforms by sequencing high-depth mRNA fragments (Kukurba and Montgomery, 2015). Most genes in vertebrates undergo alternative splicing events, resulting in different protein sequences. These splicing events regulate mRNA stability and localization (Merkin et al., 2012). Such differences are also present in interstitial tissue, and may be selectively controlled between tissues (Wang et al., 2008). We examined 3,133 CGIs identified from 20 different tissues from one chicken sample which could affect downstream genes and calculated statistically significant changes in their relationships. Analyzing these CGI and gene pairs, 121 significant units were statistically correlated. Among those, six genes (*CLDN3, DECR2, EVA1B, NME4, NTSR1*, and *XPNPEP2*) were discovered to be highly significantly correlated, and their expression was changed by alteration of DNA methylation.

## Materials and Methods

### Sample Preparation

Ogye chicken (object number: 02127) used in this study was obtained from the Animal Genetic Resource Research Center of the National Institute of Animal Science (Namwon, South Korea). Twenty tissues were dissected from an 8-month-old female chicken (breast, liver, bone marrow, fascia, cerebrum, gizzard, matured, and immatured egg, cockscomb, spleen, cerebellum, gallbladder, kidney, heart, uterus, pancreas, lung, skin, eye, and shin skin) for the RNA-seq and RRBS library preparation. Protocols for the care and experimental use of Ogye chicken was reviewed and approved by the Institutional Animal Care and Use Committee of the National Institute of Animal Science (IACUC No. 2014-080). Ogye chicken management, treatment, and sample collection took place at the National Institute of Animal Science.

### RNA Sequencing

Total RNA was extracted from 20 different tissues using 80% EtOH and TRIzol. RNA concentration was measured using Quant-IT RiboGreen (Invitrogen, Carlsbad, CA, United States). Samples were run on a TapeStation RNA screentape (Agilent, Waldbronn, Germany) to assess the integrity of total RNA. Only high-quality RNA samples (RIN ≥ 7.0) were used to construct the RNA-seq library. Each library was independently prepared with 300 ng of total RNA using the Illumina TruSeq Stranded Total RNA Sample Preparation Kit (Illumina, San Diego, CA, United States). The rRNA was depleted from total RNA using the Ribo-Zero kit. After rRNA depletion, the remaining RNA was purified, fragmented, and primed for cDNA synthesis. The cleaved RNA fragment was cloned into (first-strand) cDNA using a reverse transcriptase and a random hexamer. After this step, second-strand cDNA synthesis was performed using DNA polymerase I, RNase H, and dUTP. The resulting cDNA fragment was then ligated with a single ‘A’ base followed by an adapter. The product was purified and concentrated by PCR to generate the final cDNA library. Libraries were quantified using qPCR as per the qPCR Quantitation Protocol Guide (KAPA Library Quantity Kit for the Illumina Sequencing Platform) and TapeStation D1000 ScreenTape Analysis (Agilent Technologies, Waldbronn, Germany).

### Reduced Representation Bisulfite Sequencing

Preparation of the RRBS library was performed according to Illumina’s RRBS protocol. First, 5 μg of genomic DNA was purified with a QIAquick PCR purification kit (QIAGEN, Hilden, Germany) and digested with the MspI restriction enzyme using the TruSeq Nano DNA Library Prep Kit (Illumina, San Diego, CA, United States) and was subsequently used for library preparation. The eluted DNA fragment was terminally repaired, and the 3′ end was extended to A and ligated with a TruSeq adapter. After binding was assessed, products with lengths of 175–225 bp (55–105 bp insert DNA plus 120 bp adapter) were plated on a 2% (w/v) low-range ultra-agarose gel (Bio-Rad, Hercules, CA, United States) and purified using the QIAquick gel extraction protocol. Purified DNA was converted to bisulfite using EpiTect Bisulfite Kit (Qiagen, 59104). The bisulfite-converted DNA library was amplified by PCR (four cycles) using PfuTurbo Cx DNA polymerase (Agilent, 600410). The final product was then quantified using qPCR and assayed using Agilent Technologies 2200 TapeStation assay (Agilent, Waldbronn, Germany). Final products were sequenced using a HiSeq 2500 platform (Illumina, San Diego, CA, United States).

### Data Processing and Quantification

For integrative data analysis of the Ogye chicken transcriptome and methylome, we used one tissue associated with reproduction (uterus), two tissues at different stages of development (immatured and matured eggs), and 17 other tissues (cerebellum, gall bladder, kidney, heart, pancreas, lung, skin, eye, brisket, shin skin, liver, bone marrow, fascia, cerebrum, gizzard, and cockscomb), generating 20 pairs of each RNA-seq data and RRBS data. The Ensembl genome browser was used for its reference genome and annotation of the chicken (Galgal4, ver78 used for this study). The quality of the RNA-seq library and RRBS library was confirmed using FastQC (v0.11.5) (Andrews, 2010). In the RNA-seq library, the front 13 bp failed base quality test and so were trimmed preceding further analysis. The RRBS sequence libraries satisfied the quality score.

In total, 16,752 gene structures with annotations known in the reference genome were used for the RNA-seq data processing. The mapping rates of the kidney, pancreas, and bone marrow were less than 50%, and the highest one was for brisket (Supplementary File S1: Table S1). For the RRBS reads, Bismark was used for mapping and quantifying DNA methylation level (Krueger and Andrews, 2011). On average, 52% of the reads were uniquely mapped, and the reads covering the CpG area were 12.6× on average (Supplementary File S1: Tables S2, S3). All reads including six samples (gall bladder, pancreas, shin skin, liver, cocks comb, spleen) with an average depth lower than 10× were discarded preceding further analysis (Supplementary File S1: Table S3).

Reference sequence alignment was performed using Tophat2 (TopHat v2.0.13) for transcriptome quantification. The transcriptome assembly for each sample was held by cufflinks (v2.2.1) (–GTF-guide –frag-bias-correct –multi-read-correct) and differential expression of the transcripts were quantified in fragments per kilobase million (FPKM) using cuffdiff (v2.2.1). The expression patterns of each tissue in PCA and MDS were visualized by CummeRbund (v0.1.3) (Trapnell et al., 2012). We constructed a matrix using an in-house R (v 3.1.2) script and compared the differences between the tissues using ensemble gene names. The genes which were not equal to FPKM row sum 3 were filtered.

Converted reference sequence alignment of the RRBS libraries was performed for DNA methylome quantification. In the RRBS libraries, the methylated sites of cytosines and thymines were transformed into the CpG sites and were aligned to the reference sequence using bismark_genome_preparation, a part of bismark (v0.14.5). After the alignment of the methylated sites the quantification was performed using bismark_methylation_extractor. Extracted information on the converted cytosines from the mapped reads were converted into a bed format. An in-house program was used to calculate DNA methylation percentages of CpG and non-CpG sites. All regions with less than 10× site coverage were discarded. The 21,664 CGIs described in the reference genome were then used to identify the promoter, gene body, CGIs, and CGI shores along with Ensembl gene annotation and CGI annotation using the UCSC genome browser^1^. The promoter region was set as 2 kbp upstream based on the transcription start site (TSS), gene body region as the TSS to transcription end site (TES). The CGI shore was set to be 2 kbp upstream and downstream of the CGIs. Statistical calculations were performed using Bedtools (v2.26.0-112) for different genomic features. Quantification of CGIs were calculated by taking the median value of DNA methylation levels using Seqmonk (Andrews, 2007).

### Gene Expression Patterns and Tissue-Specific Genes

Expression patterns from the 20 tissues were calculated using Euclidean distance and ward linkage based K-means clustering with a quantified FPKM matrix. Clustering results showed that 12 groups (*K* = 12) reached a positive correlation (*r* > 0.5) with reference to the correlation plot (Supplementary File S1: Figure S1). To best represent the characteristics of each cluster, the tissues were compared with each other and genes were selected that satisfied the fold change criteria in at least more than one pair. The fold change value was calculated using a range from 2 to 256, and a fold change of 16 was selected to satisfy the above criteria (Lin et al., 2014). Finally, 1,492 selected genes were filtered and transformed into standard *z*-scores to visualize as a heatmap. A GSEA to analyze functional annotation in each of the clustered genes was performed using PathwayStudio (Elsevier, Amsterdam, Netherlands). The gene list within each cluster was used identify interactions by using network analysis denoting direct protein regulation in order to perform an entity list summary. The GO terms were identified based on highest enrichment scores and their expression of characteristics in each cluster. Finally, visualization of the boxplot with the gene expression distributions was combined using R package ComplexHeatmap (Gu et al., 2016).

### Characteristics of Gene Expression Patterns

Twenty tissues were compared pairwise to identify DEGs. To compare differences within pairs the cutoff for significantly altered genes was set at a *p*-value of ≤0.05. A pairwise comparison table was constructed centered on the DEG counts, and was visualized over the network of tissues to make comparisons easier. Network nodes represent the total sum of DEGs in a given condition, and edges represent DEGs compared between two tissues. DEGs that appeared between similar tissues were used for GSEA, to explain the difference in tissues.

### Genome-Wide Detection of Alternative Splicing

Predicted transcripts were formed based on Cufflink reference annotation based transcript (RABT) assembly and were examined by cuffcompare (v2.2.1) using 130,295 exons. The inclusion ratio (ψ) of exons for each tissue was calculated by rMATS (Shen et al., 2014) and in-house scripts. Regions of read coverage below 10 were discarded after combination of paired-end read information and read split information. The 2,910 canonical exon splice sites were used to create an exon usage matrix with missing data in all tissues (Burset et al., 2000). Then, exons with exon exclusion (ψ ≤ 0.3, ψ ≥ 0.7) in different tissues were used to find switch-like exon candidates. A number of 160 switch-like exon candidates were examined and functional classification was carried out using DAVID (Dennis et al., 2003). The 160 switch-like exon candidates were then converted to refSeq ID; among them 63 matched the results of the DAVID calculation and only seven genes were enriched (enrichment score 3.18; Supplementary File S1: Table S9). Differential exon usages (DEUs) between samples in different developmental stages (matured eggs and immatured eggs) were calculated by rMATS. We then made a genome-wide circular plot of switch-like exon candidates, inclusion ratios of the 20 tissues, and differential values of exon usages at developmental stage tissues using the R package omicCircos (Hu et al., 2014).

### Characteristics of DNA Methylation and Pairwise-Comparison Among the 20 Tissues

Pair-wise comparisons of differential methylated sites (DMS) were calculated using MethylKit (1.0.0) in all conditions (FDR ≤ 0.05) (Akalin et al., 2012). To find DNA methylation differences between tissues, a network using DMS counts across tissues was constructed. The 2 kbp region upstream of the TSS was designated as a region of CGIs using bedtools. The median value of DNA methylation sites in the CGIs was defined as a representative value of all the CGIs. Based on the gene expression matrix, we performed network analysis using 3,133 CGIs present in the promoter regions. Unsupervised clustering was performed using the Markov cluster algorithm of BioLayout Express 3D (v3.2) (MCL = 1.3) (Theocharidis et al., 2009). We confirmed that three subnetworks appeared and performed GSEA. In-house python scripts were used to generate matrices using DNA methylation level of CGIs and FPKM of downstream genes. Correlation between the matrices was examined by Pearson’s correlation test. Among the pairs of CGIs and downstream genes, 121 significantly negatively correlated units were found (*p*-value ≤ 0.05). We performed GSEA using Pathway Studio for the downstream gene names of the selected 121 units. Then, we selected six genes with high significance (Pearson’s correlation tests; *p*-value ≤ 0.001). We showed relationship between gene structure and CGI using the IGV (Win_2.4.19) (Robinson et al., 2011) (Supplementary File S1: Figure S7).

## Results

### Gene Expression Patterns and Tissue-Specific Genes

**FIGURE 1:**
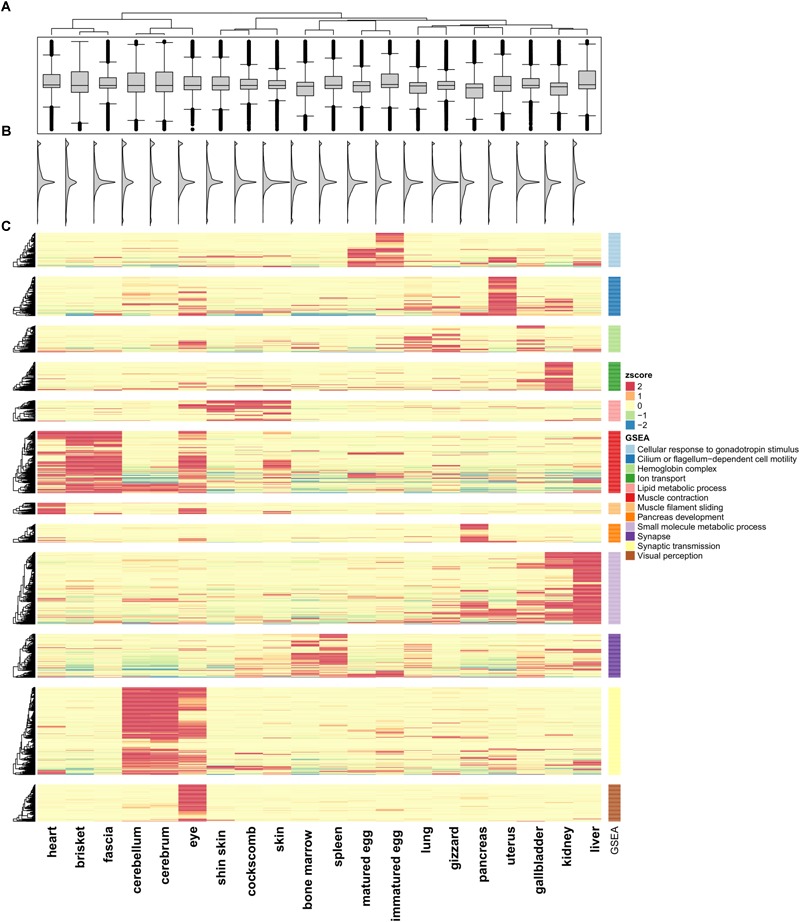
Unsupervised K-means clustering of TSGs. **(A)** Gene expression differences in the 20 tissues. **(B)** Smoothed histogram of gene expression values. **(C)** Heatmap of tissue-specific genes using k-means clustering (*k* = 12; Euclidian distance method; ward linkage method). FPKM values in each of the tissues were converted to *z*-scores. The GO terms for each cluster used the term with the highest enrichment score. The cluster names are labeled 1–12 from top to bottom.

We quantified RNA-Seq reads from 20 tissues to observe changes in gene expression patterns within an individual chicken model. FPKM values were calculated for quantification of RNA-seq samples (Trapnell et al., 2012). Based on Ensembl gene annotation, we created a matrix for each of the 20 tissues. Pearson’s correlation test was performed using 16,752 genes (Supplementary File S1: Table S4) and a positive correlation was detected in all paired sets (*p* < 2.2e-16). The highest positive correlation was between the cerebrum and cerebellum (*r* = 0.819; *p*-value < 2.2e-16), and the pair of tissues with the weakest correlation was the eyes and liver. Tissue pairs with a strong positive correlation (*r* > 0.5) included the heart, brisket, and fascia, and the *r*-values of the cockscomb, skin, and shin skin were high. Especially in different developmental stage tissues, matured eggs and immatured eggs, showed a strong positive correlation (*r* = 0.638; *p*-value < 2.2e-16) compared with other tissues. The correlation matrix was then used for a clustering method in which tissues with strong positive correlations (*r* > 0.5) were considered together. Here, a total of 12 clusters were evident (Supplementary File S1: Figure S1). The distribution of gene expression displayed two peaks and we were able to observe a small peak in all tissues under FPKM 1 (Figure 1B). The median values of the distributions considered as the main peaks were similar (Figure 1A,B).

To investigate the similarity between tissues and characteristics among the functional genes, significantly different expression levels according to the conditions were identified (fold change ≥ 16). Unsupervised K-means clustering (*k* = 12) was used to select 1,492 TSGs and the number of genes in each cluster was 60–200 (Figure 1C). The patterns for the cerebrum and cerebellum (which had the strongest correlation) were found to be similar. In the eye, two kinds of patterns appeared, an independent one and shared patterns from clusters 6, 11, and 12. Two cluster groups from 6 and 11, which were groups from muscle and brain tissues, shared patterns different from other groups. Unlike the other tissues, the gene sets for the uterus had high expression values in only one cluster, and had low values for all of the other clusters. There were six clusters with similar patterns in more than two tissues (clusters 1, 5, 6, 9, 10, 11). Among them six clusters showed a strong positive correlation when combining *r* > 0.5 samples, with the exception of cluster 9 (liver and kidney), which showed high expression values but a low correlation value (*r* = 0.471). Cluster 1 contained immatured and matured egg tissue classed as in the developmental stage. For the heart, brisket, and fascia, the results indicated that the tissues had evident characteristics of muscle fiber (cluster 6). Similarly, in cluster 5, the shin skin, cockscomb, and skin showed relatively high expression values. In clusters 9 and 11, the kidney and liver showed a single expression value, and bone marrow and spleen showed particularly high expression values. All grouped clusters shared similar cytological functions.

Gene set enrichment analysis was performed to investigate the more detailed functions of the gene sets in each cluster (Supplementary File S1: Table S5) (Subramanian et al., 2005) using Pathway Studio (v.11.2.04, v11.4.08, Elsevier; Mammal database). Cluster 1 contained tissues in the developmental stage, and consistent with this, many genes related to gonadotropin stimulus were found, such as the early growth response (EGR) and steroidogenic acute regulatory protein (*STAR*) family, associated with steroid hormone synthesis. In particular, forkhead box L2 (*FOXL2*) associated with ovarian development had the most abundant relationship (Borman et al., 2004). Cluster 5 included skin tissue were related to fatty acids. For the bone marrow and spleen tissue, the terms associated with blood and cytokine receptor activity, associated with immune system were the most enriched. Cluster 12, which had high expression value only in the eye, had enriched terms including those related to visual perception and photo transduction. This cluster contained many genes related to neural differentiation such as nuclear receptor subfamily 2 group E (*NR2E3*) and orthodenticle homeobox 2 (*OTX2*) and particularly on cone rod homeobox (*CRX*), which controls the differentiation of photoreceptor cells.

### Characteristics of Gene Expression Patterns Among Tissues

**FIGURE 2:**
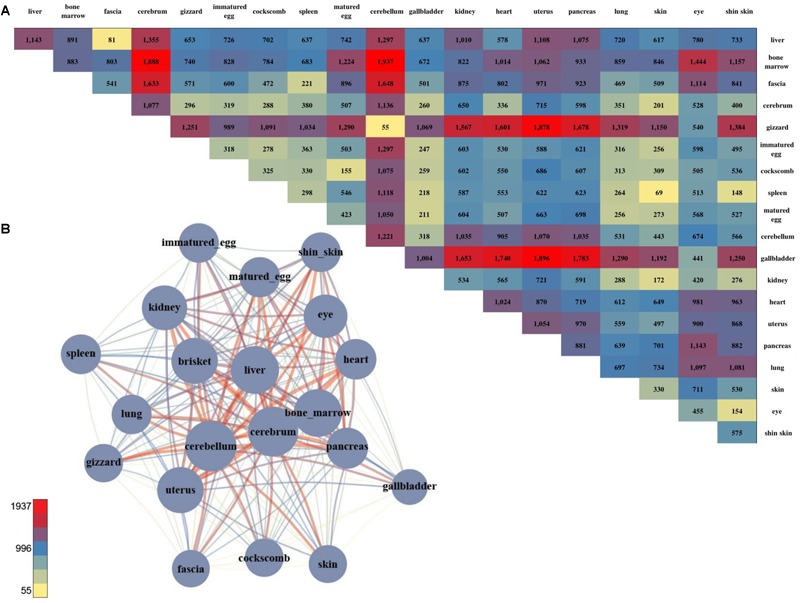
The number of DEGs and the pair-wise comparison of the 20 tissues. **(A)** The number of DEGs that were compared with each other among the 20 tissues. **(B)** Network created using the DEGs. The total number of DEGs in a given condition is represented by a node, and the number of DEGs in the condition is indicated by an edge.

We successfully found gene sets that were highly expressed in specific tissues and identified groups of tissues that shared the same gene set. However, there were distinct functional differences among the tissues, and DEGs were examined to investigate these differences. 66,133 DEGs were examined by combining all of the sample together (Figure 2A; negative binomial tests; *p*-value ≤ 0.05). Among the pairwise comparison of 20 tissues, the cerebellum and liver had the highest number of statistical differences, and the cerebrum and cerebellum had the strongest positive correlation and lowest number of DEGs. Also, two samples of brain tissue revealed a relatively large number of DEGs when compared to other tissues (Figure 2A).

For a more intuitive comparison, we presented the DEG counts of the paired samples in a network (Figure 2B). The tissues with a relatively small number of DEGs were the cluster that included the brisket, heart, and muscle of fascia and the cluster that included the skin, shin skin, and epithelial tissue of the cockscomb. Samples with relatively low DEG counts were near-positioned or highly correlated samples determine by k-means clustering. To identify differences between these tissues, we identified genes that exhibited statistical differences in clustered tissues (*p*-value ≤ 0.05). Kidney and liver pairs had the most DEGs, and the cerebrum and cerebellum, which had the highest *r*-values, had the lowest number of DEGs. The brisket, heart, and fascia including muscle had a higher number of DEGs than clusters compared with other tissues. A comparatively small number of DEGs, between 40 and 60, were observed in the cluster of skin, cockscomb, and shin skin including skin. The lowest number of DEGs were detected in pairs of matured eggs and immatured egg, and also for the cerebrum and cerebellum, which had the strongest correlation (Supplementary File S1: Table S4).

Gene set enrichment analysis was performed to determine the biological significance of the DEGs of clustered tissues (Supplementary File S1: Table S6). The most enriched term in the bone marrow and spleen pairs was extracellular matrix organization in which the collagen gene appeared most abundantly. The collagen gene is the most common protein in multi-cellular animals, accounting for 30% of the extracellular matrix (Daley et al., 2008). Since the kidney and liver pairs showed the largest difference, genes with the terms synaptic transmission and ion transport pathway commonly appeared.

### Genome-Wide Detection of Alternative Splicing

Researchers in the splicing regulation field have utilized comparative approaches to reveal tissue-specific or disease related alternative splicing (AS) events (Heinzen et al., 2008; Buljan et al., 2012). To investigate AS events, the inclusion level of alternative exons was quantified in several studies using a transcriptomic quantification method (Pan et al., 2008; Wang et al., 2008). We examined the inclusion level of alternative exons by quantifying the percentage of the number of reads that match the two splice junctions formed by exon inclusion, over the splice junction formed by exon skipping. Splice junction reads were used for quantification of minor isoforms with different frequencies, as a function of the read coverage or RPKM.

**FIGURE 3:**
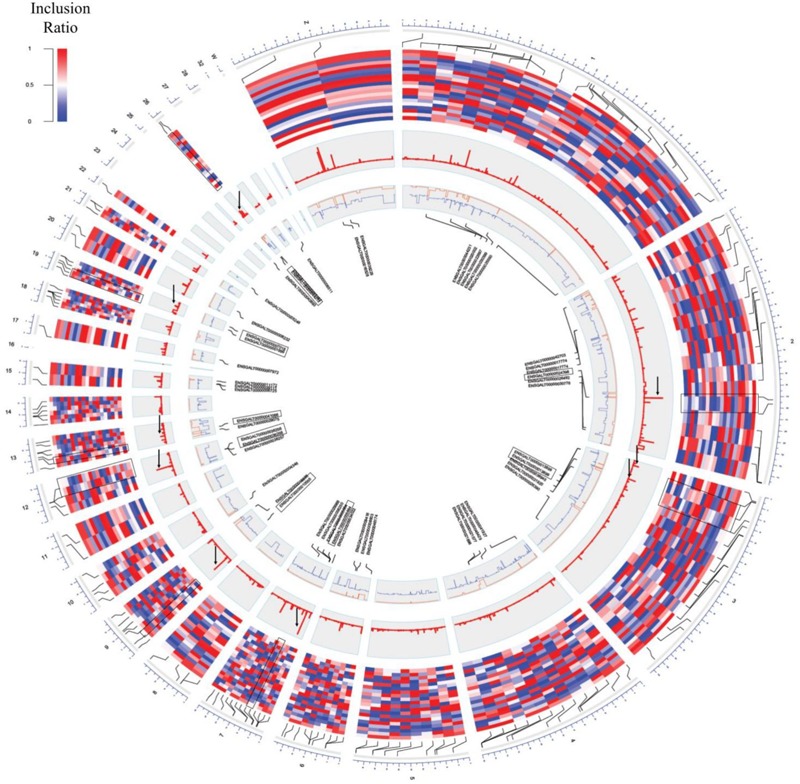
Circos plot of switch-like exons. From the outer circle to inner circle are the genomic position, inclusion ratios, developmental stage, significance signal [–log(*q*-value)], inclusion ratios of the immature eggs and mature eggs (red: mature eggs, blue: immature eggs), and the gene name of the significantly changed exons. The highlighted pairs are the genes that were converted and for which GO terms were found (black box). Significantly changed signals are marked with a black arrow.

The inclusion ratios for individual tissues were calculated using rMATS to identify exons used only in specific tissues (Shen et al., 2014). The inclusion ratio of 130,295 exons was calculated using transcripts made with cufflinks. A total of 22,482 (17.25%) exon skippings, 6,859 (5.26%) alternative 3′ exons, and 4,188 (3.21%) alternative 5′ exons were found by filtering all of the sites where the number of reads of the splicing sites did not reach 10× coverage (Supplementary File S1: Table S7). We found 2,910 exon skippings, 669 alternative 3′ exons, and 479 alternative 5′ exons including exon skipping events that used canonical splice sites. Switch-like alternative splicing exons indicate high-level usages in some tissues but low-level usages in other tissues (Wang et al., 2008). To provide candidates for switched-exons and to catalog alternative exons that vary among the tissues, 160 exon skipping events with an inclusion ratio of ≤0.3 and ≥0.7 were selected in at least one tissue (Figure 3, second outer circle). Seventy genes were enriched with GSEA using 160 alternative splicing candidates. Thus, among the 160 alternative splicing of enriched genes, seven genes were significant in immatured egg and matured egg related to developmental stage (DAVID Enrichment score 3.18; Supplementary File S1: Table S9). The splicing factor has the largest enrichment score. We then tested DEU in immature and mature eggs, and identified 82 significantly changed exons out of 6,726 exon skipping events (Figure 3, fourth outer circle, Supplementary File S1: Table S8; FDR ≤ 0.05). GSEA analysis using 39 genes mapped out 82 exons, revealing terms related to mitosis and histone demethylation (Supplementary File S1: Table S10). We observed eight significantly enriched genes in autosomal chromosomes in tissues related to the developmental stage, and we also observed significance in genes present in sex chromosomes but not in enriched terms (Supplementary File S1: Table S9 and Figure 3, third outer circle).

### Characteristics of the Differences in Pairwise-Comparison of DNA Methylation

We next quantified the RRBS data to distinguish the epigenetic functions of DNA methylation and to measure the diversity occurring within an individual. The RRBS data was used to discriminate CpG sites from CHG sites and determine the percentage of methylation in DNA methylation sites. Approximately 70% of methylation events in animal genomes are known to occur in CpG sites (Law and Jacobsen, 2010). We focused on the CpG sites and calculated the DNA methylation levels in the reliable (read depth ≥ 10) 27 Mbp promoter region and the 405 Mbp gene body region. Using the CGI definition provided by UCSC genome browser (see text footnote 1), 21,664 DNA methylation sites of CGIs were obtained using 13,777 promoters and gene bodies. In the case of DNA methylation sites in CGIs, the percentage of hypo-methylation in the gene body was higher than that of hyper-methylation in most samples. The rates of hypo-methylation in all samples were high in the promoter region (Supplementary File S1: Figure S2), and there were few hyper-methylated DNAs in the CHG region. In the sites that were computable, there were ∼6 times more CHG sites than CpG sites.

Calculation of the DNA methylation of CpG sites was estimated using the methylation level of 21,664 CGIs without missing data. Pearson’s correlation tests were performed using the matrix and all tissues presented positive correlations (Supplementary File S1: Figure S3). Liver and spleen data with an average depth of less than 5× displayed a weak correlation compared to other tissues. The tissues presenting the strongest correlation were the cerebellum and cerebrum. In addition, tissues organized by organ system category were located close to the dendrogram (data not shown). We analyzed the correlations of tissues through paired correlation analysis (PCA), and all tissues except the spleen and liver were in moderately close positions (Supplementary File S1: Figure S4A). The density intensity decreased as the methylation level increased, because the ratio of hypo-methylation was higher in all tissues, but a sub-peak was observed around 50% aside from the hyper-methylation peak (Supplementary File S1: Figure S4B). We used a gene structure to draw a trend plot of DNA methylation level 2 kbp upstream and 2 kbp downstream (Supplementary File S1: Figure S4C). The patterns here were almost the same but differences occurred according to the read depth. On the basis of the TSS, the methylation level decreased, then increased, and decreased again at the TES: a common pattern that can be found in several other studies (Cokus et al., 2008; Guo et al., 2015).

**FIGURE 4:**
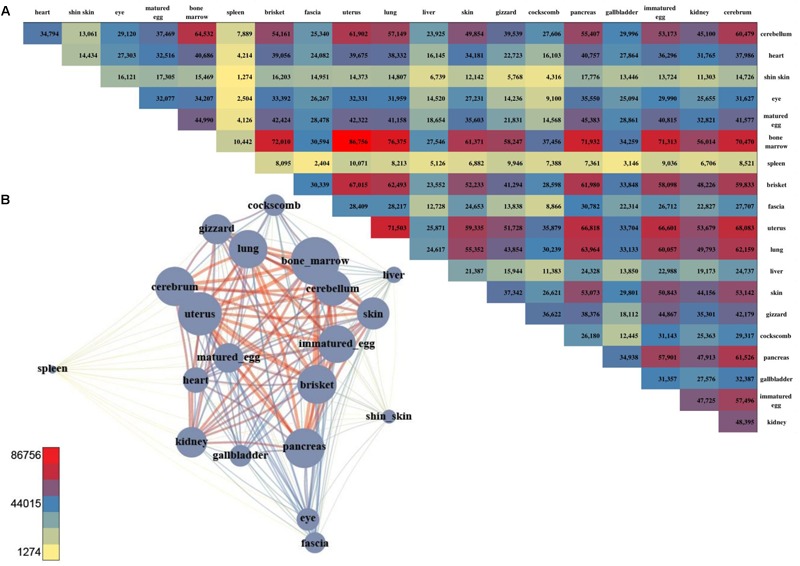
The number of DMS and the pair-wise comparison of the 20 tissues. **(A)** The number of DMSs that were compared with each other among the 20 tissues. **(B)** Network created using the DMSs. The total number of DMSs in any pair-wise condition is represented by a node, and the number of DMSs in the condition indicated by an edge.

For DMSs, DNA methylation differences among the tissues were computed, and were counted site by site for quantification. The tissues with the least difference were the cerebellum and cerebrum, and 112 DMS were found to be significantly different (Figure 4A,B). The largest difference between the tissues was found between the uterus and bone marrow, with 74,121 DMSs. After pairwise comparison across the tissues, the largest number of the DMSs occurred with in the bone marrow, showing in 2,240–74,121 DMSs when compared with other tissues. The average deviation was 34,887, and the standard deviation was 20,745, demonstrating that the fluctuation was large compared to other tissues. The fewest DMSs were observed in the spleen and shin skin according to a low average depth.

### Correlation Across Tissues Between Expression Level and DNA Methylation

**FIGURE 5:**
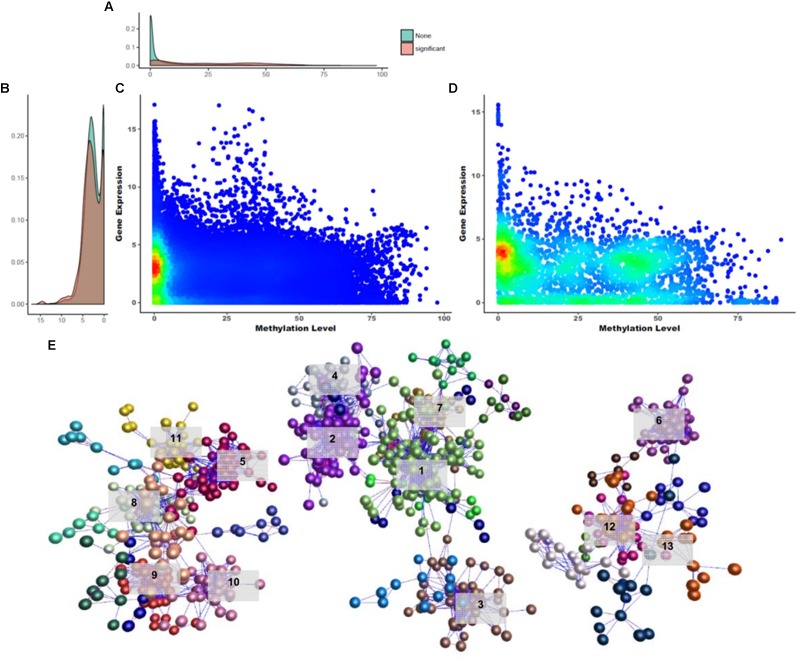
Overview of gene silencing DNA methylations. **(A)** Gene expression density in *Y*-axis. **(B)** DNA methylation density in *X*-axis. **(C,D)** Density distribution of GSM candidates and GSMs. **(E)** Network constructed using GSM candidates (BioLayout Express 3D; *r* > 0.85; MCL = 1.3). It was divided into three subnetworks and 35 subgroups. The top 13 subgroups that had peaks in one tissue are marked. Highlighted edges (red) are significantly correlated genes with gene-silencing methylations.

Previous studies have demonstrated clearly the mechanism of gene expression regulation through DNA methylation (Nan et al., 1993; Hark et al., 2000; Jones, 2012) and these mechanisms are known to be important during developmental stages and differentiation (Bird, 2002). Based on this fact, we tried to identify the relationships between DNA methylation changes and gene expression across different tissues. To do this, we compared promoter methylation levels with FPKM using 13,777 gene annotations to determine how the level of DNA methylation changes affects gene expression. First, quantified matrices of the DNA methylation level and gene expression were examined. We constructed 3,133 units around the CGIs in the promoter regions to determine correlations between DNA methylation levels and gene expression (Supplementary Data Sheet S1). A negative correlation between DNA methylation level and gene expression pairs was evident (Figure 5A; Pearson correlation test; *r* = -0.0837). Most of these distributions showed a uniform density, but in the hypo-methylated region, many pairs were clustered between 0 and 5 FPKM. To identify candidates that could be involved in gene silencing, 121 gene silencing DNA methylations (GSMs) and their downstream gene expressions were determined as pairs with a negative correlation (*r* < 0; *p*-value ≤ 0.05) (Supplementary File S1: Table S11). The distribution of 3,131 CGI and the 121 GSMs differed little in gene expression; however, there was a difference in their DNA methylation level. Gene expression density was very high in genes with no significance in regions below FPKM 1, but close to uniform when density was measured using only 121 GSMs (Figure 5A). On the other hand, the density of DNA methylation levels was high in hypo- and hyper-methylated DNA and relatively low in the intermediate level (Figure 5B). Hypo-methylated DNA density was higher than hyper-methylated DNA density (Figure 5C,D). Regardless of whether GSMs were selected or not, their distribution showed a negative correlation. And the GSM density of hypo-methylated regions with gene expression values of 4–5 was particularly high.

A DNA methylation network analysis was performed to investigate the interaction of 3,133 GSM candidates (Figure 5E; BioLayout Express 3D; *r* > 0.85; MCL = 1.3). Here, 29 subgroups were constructed and connected into 13 clusters. A total of 829 CGIs remained, and the subgroup belonging to clusters with one peak contained 653 CGIs. The GSEA of the downstream genes of CGIs were composed in three subnetworks, and from the left, the 213, 331, and 109 CGIs appeared as unconnected bundles. In the first subnetwork, the cockscomb, gallbladder, gizzard, eye and bone marrow were combined in one peak. In this subnetwork, the Rab-GAP TBC domain, nuclear pore, and transmembrane protein 41 (*TMEM41)* family were found as enriched terms (Supplementary File S1: Figure S5A). The Rab-GAP TBC domain has several functions well known in membrane trafficking (Gillingham and Munro, 2003; Barr and Lambright, 2010; Müller and Goody, 2018). The nuclear pore and *TMEM41* family were the next most enriched terms, as all three were associated with the cell membrane. The second subnetwork was the largest, composed of the spleen, liver, shin skin, skin, and cerebrum (Supplementary File S1: Figure S5B). Terms related to protein degradation and glucose metabolism were observed in this network. The last subnetwork showed the smallest peak for cerebellum, immature egg and mature egg (Supplementary File S1: Figure S5C). Finally, we analyzed the GSEA of the downstream genes using 121 significantly correlated CGIs. Among the biological processes, terms related to liver development and cardiac morphogenesis were observed.

**FIGURE 6:**
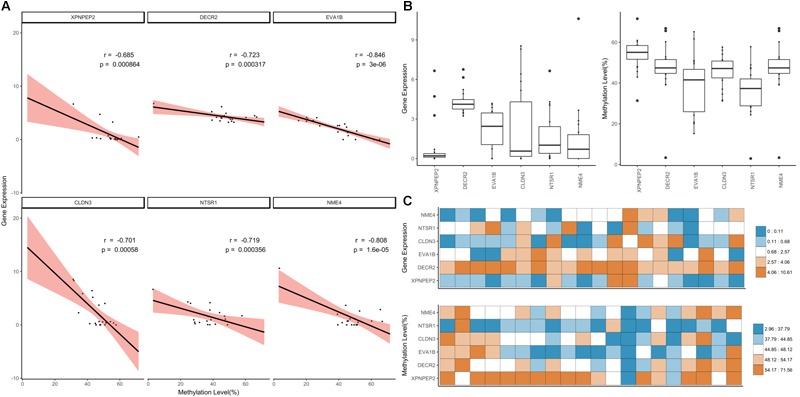
Downstream genes of highly significant GSMs. **(A)** Correlation of the highly significant GSMs and distribution of DNA methylation and gene expression values. The simple linear correlation and 95% confidence intervals marked using the lm function (black line, red range). **(B)** Distribution of gene expression and DNA methylation values was expressed using boxplot. **(C)** Heatmap of gene expression and DNA methylation values.

We applied a hard filter to the GSMs and finally selected six genes (*CLDN3, DECR2, EVA1B, NME4, NTSR1* and *XPNPEP3*) associated with epigenetic regulation, differentiation and cancer (Figure 6; Pearson’s correlation test; *p*-value ≤ 0.001). Most DNA methylation levels were moderate and gene expression levels were between 0 and 10 when converted to log_2_ (FPKM+1) (Figure 6A,B). In particular, the expression level of *NME4* was high in the liver (Supplementary Data Sheet S2; FPKM: 1558.41). *XPNPEP2* had the lowest gene expression distribution, but highest methylation level in most tissues (Figure 6C). *XPNPEP2* is located on the X chromosome, which possesses hydrolase and aminopeptidase activities. This gene plays a role in the metabolism of the vasodilator bradykinin, and is associated with colorectal cancer through somatic methylation alterations (Navarrete-Meneses and Pérez-Vera, 2018). The gene with the largest expression change was *CLDN3* (Figure 6B) and a change in DNA methylation level was concentrated at around 50%. A previous study identified that the promoter of *CLDN3* is affected by epigenetic processes through DNA methylation in ovarian cancer cells (Honda et al., 2007). *DECR2* had relatively high gene expression values (Figure 6B). The gene regulation of lipid metabolism by peroxisome proliferator-activated receptor alpha (RPARalpha) has been reported to be a candidate in prostate cancer development, that function by regulating gene expression through DNA methylation (Yang et al., 2013). *EVA1B* was the most significant negatively correlated gene, with the highest variation of DNA methylation. In the bone marrow, brisket, cerebellum, cerebrum and uterus, *EVA1B* had relatively high DNA methylation levels (Figure 6C), which is known to regulate programmed cell death as the paralog gene of *EVA1A*. Epigenetic regulation of this gene was previously reported using a dental pulp stem cell model, in which a hyper-methylated DMR region in this gene was reported with embryonic stem cells (Li et al., 2016; Dunaway et al., 2017). *NTSR1* had the lowest median DNA methylation level (Figure 6B), where DNA methylation levels were relatively high in the brisket and kidney (Figure 6C). *NTSR1* belongs to the superfamily of G-protein coupled receptors, and its signaling has been determined to activate downstream MAP kinases and prevent apoptosis. DNA methylation of this gene has been studied in pancreatic, colorectal and ovarian cancers (Hagihara et al., 2004; Nelson et al., 2012; Kamimae et al., 2015; Navarrete-Meneses and Pérez-Vera, 2018), and it is known to be a general marker of epigenetic regulation. Finally, *NME4* showed a significant negative correlation and had a pattern similar to *NTSR1*, with a particularly high gene expression value and low DNA methylation level in the liver. DNA methylation levels of *NME4* were slightly higher than those of *NTSR1* (Figure 6B). This gene encodes ubiquitous enzymes that catalyze the transfer of gamma phosphatases, and was identified as an epigenetic biomarker through methylation microarray-based scanning in a colorectal cancer cohort (Mori et al., 2011). All six of the gene-silencing DNA methylations (GSM) were relatively low in liver (Figure 6C), and all of these genes have previously been reported to regulate downstream genes by altered DNA methylation in their promoters.

### Investigation of the Relationships of Imprinted Genes to Infer the Epigenetic Function of GSMs

In chickens, there are no known imprinted genes, however, we investigated known or predicted genes in other species to identify the epigenetic functions of 121 GSMs (Plasschaert and Bartolomei, 2014; Choi et al., 2015). We searched for information in the imprinted genes database and found three genes that overlapped at Geneimprint^2^ (Supplementary File S1: Table S12). There were two predicted genes in humans (*FOXG1, PRDM16*) and one imprinted gene in the gray short-tailed opossum (*MEIS1*) (Luedi et al., 2005, 2007; Douglas, 2013). In the case of *FOXG1*, the methylation level in the cerebrum and cerebellum was drastically decreased, and that of FPKM was found to be high (Supplementary File S1: Figure S6A). *FOXG1* has been reported to play an important role in brain development and is evolutionary conserved in vertebrates. Its expression was compared with that of the human gene using BioGPS (Wu et al., 2009) (Supplementary File S1: Figure S6B). *MEIS1* was found to be highly expressed in the uterus and gizzard. This gene is known to function as an enhancer to regulate the cofactors of the *HOXA10* homeobox gene. *HOXA10* is a gene that regulates embryonic uterine development and endometrial receptivity (Xu et al., 2008). Compared with that in humans, high expression was observed in the uterus. For *FOXG1* and *MEIS1*, there were two tissues with relatively high gene expression values, but *PRDM16* was high only in the pancreas. *PRDM16* encodes a transcription factor and histone H3 methyltransferase, and a genetic screening study reported that it plays an important role in the development of the pancreas (Sugiyama et al., 2013). In BioGPS, no significance were found to be specifically highly expressed, and expression values in the heart and liver were relatively high. Among *FOXG1, MEIS1* and *PRDM16*, the methylation level of *PRDM16* was high on average (Supplementary File S1: Figure S6C). In fact, *FOXG1* and *MEIS1* had identifiable tissue-specific properties.

## Discussion

In the field of epigenetic studies, NGS technology has brought many advantages and advances (Hurd and Nelson, 2009; Schweiger et al., 2011). In particular, it is possible to produce more accurately quantified results by using high throughput data and observing changes in the base-pair resolution of the genome. We produced data on two different platforms and quantified the transcriptome and DNA methylome through RNA-seq and RRBS, respectively. This data allowed us to observe even very small differences in DNA methylation and gene expression. Based on this, we sequenced as many tissues as possible and investigated the relationship between them. Previous DNA methylome studies were identified that used embryonic stem cells or samples in the developmental stages (Laurent et al., 2010; Spiers et al., 2015; Fu et al., 2018). In addition, there have been studies that considered difficulties in finding statistically significant results due to a lack of sample diversity (Habibi et al., 2013; Chen et al., 2015; Choi et al., 2015; Zhou et al., 2016). Here however, we discovered significant results because we examined the relationship between DNA methylation and gene expression using 20 different adult tissues.

**FIGURE 7:**
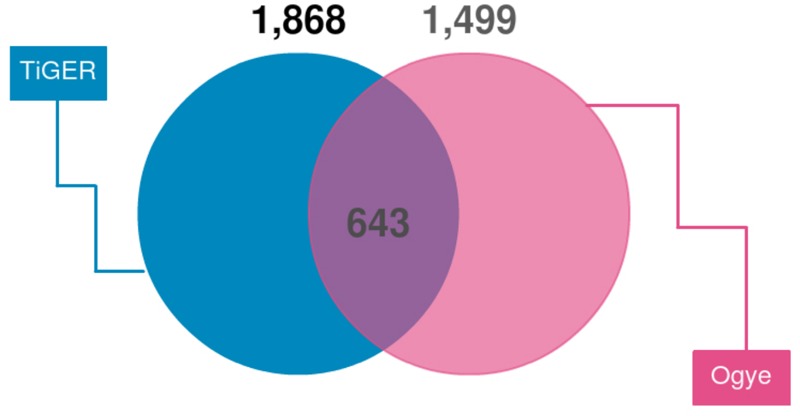
Venn diagram of TSGs intersecting with human TSGs (TiGER database). TiGER database genes were selected with paralog genes of the chicken gene annotations. Only single copy genes were used.

First, we looked for a set of highly expressed genes in specific tissues to examine the patterns of gene expression. This not only helped to understand the features of the tissues but also to understand the relationships between them (Figure 1, 2). The gene sets found reflect the characteristics of each tissue. To compliment this, many of these genes are found in the human TSGs database TiGER^3^ (Liu et al., 2008) (Figure 7). One cluster, however, did not represent a single tissue, which explains the similarities in expression pattern across similar tissues (Figure 1). In the case of the eyes, however, the muscle-related tissues and gene sets that were associated with muscle contraction and muscle filament sliding were shared. When we searched for human eye TSGs in the TiGER database, we found that many of them overlap with muscle TSGs (14/232). This suggests that the eye tissue can have both attributes. In brief, we identified both gene sets that are specifically expressed in only one tissue and genes that are expressed in similar patterns in tissues with similar functions.

The relationship between CGIs in promoter regions and downstream genes they may regulate has been studied for a long time (Schilling and Rehli, 2007; Haberle and Lenhard, 2016). However, there have not been many studies undertaken on a genome-wide level. Previous studies have found a way to measure tissue-specific DNA methylation and its effects (Chen et al., 2015; Choi et al., 2015; Zhou et al., 2016). On the other hand, we assessed the relationship between DNA methylation and gene expression at the genome-wide level and found clear correlations between them. In our study, we found 121 statistically significant GSMs, related to genes associated with development or human disease. Finally, six genes were selected by hard filtering and these genes were also found to be associated with development, differentiation, epigenetic regulation, and cancer. All of these genes were found to have CGIs that significantly overlapped with the first exons (Supplementary File S1: Figure S7). In addition, *XPNPEP2, NME4* and *NTSR1* were contained in 1,492 TSGs. In particular, XPNPEP2, an X-chromosome linked gene, is known to change the state of DNA methylation depending on sex, age, and environmental conditions (Liu et al., 2010). We believe additional epigenetic studies are needed for these three genes.

## Conclusion

In conclusion, genes that display a correlation between DNA methylation and their expression in specific tissues are responsible for significant changes in not only in developmental stages but also in adult tissues. We made this finding by using tissues that are not in developmental stages, but have developed in adults. This can either represent a change that occurs at the developmental stage, or it can be the result of epigenetic changes. This study presents a methodology that can be used by other researchers using omics data combined with different types of NGS data, and provides them with the opportunity to use our transcriptome and DNA methylome landscapes.

## Ethics Statement

This study was carried out in accordance with the guidelines of the Institutional Animal Care and Use Committee (IACUC) and was approved by the National Institute of Animal Science, Rural Development Administration, South Korea (Approval No: 2014-080). Animal preparation and experiments were conducted in accordance with protocols approved under the guidance of the IACUC.

## Author Contributions

NK designed and supervised this project. W-JL and KK analyzed the data and drafted the manuscript. J-YK and SJ interpreted and visualized the results. All authors reviewed and approved the final manuscript.

## Conflict of Interest Statement

The authors declare that the research was conducted in the absence of any commercial or financial relationships that could be construed as a potential conflict of interest.

## Footnotes

1 https://genome.ucsc.edu

2 http://www.geneimprint.com

3 http://bioinfo.wilmer.jhu.edu/tiger/

## Abbreviations

CGI: CpG island
DEG: differentially expressed gene
DMS: differentially methylated site
FPKM: fragments per kilobase of exon per million mapped fragments
GSEA: gene set enrichment analysis
RRBS: reduced representation bisulfite sequencing
TSG: tissue-specific gene
WGBS: whole genome bisulfite sequencing
