# Supplementary material for: Identification of DNA-Methylated CpG Islands Associated With Gene Silencing in the Adult Body Tissues of the Ogye Chicken Using RNA-Seq and Reduced Representation Bisulfite Sequencing
Source: Front Genet. 2019 Apr 16;10:346. doi: 10.3389/fgene.2019.00346 (PMC6476954; doi:10.3389/fgene.2019.00346)
Supplement: Supplementary file 1 [file Table_1.DOCX]

**Table S1 Brief statistic results of reference sequence alignments by using RNAs-seq**

|  | **input reads counts** | |  | **Uniq mapped reads** | |  | **mapping rates** | | |
| --- | --- | --- | --- | --- | --- | --- | --- | --- | --- |
|  |  |  |  | **counts** | |  |  |  |  |
| **Tissues** | **forward** | **reverse** |  | **forward** | **reverse** |  | **forward** | **reverse** | **forward - reverse** |
| **matured egg** | 30,873,699 | 30,873,699 |  | 22,394,965 | 21,748,396 |  | 72.54% | 70.44% | 2.10% |
| **cerebellum** | 30,798,145 | 30,798,145 |  | 24,644,794 | 24,421,460 |  | 80.02% | 79.30% | 0.72% |
| **gallbladder** | 35,862,229 | 35,862,229 |  | 22,645,815 | 22,541,579 |  | 63.15% | 62.86% | 0.29% |
| **kidney** | 29,953,007 | 29,953,007 |  | 13,260,148 | 13,090,924 |  | 44.27% | 43.70% | 0.57% |
| **heart** | 30,986,431 | 30,986,431 |  | 24,876,592 | 24,969,026 |  | 80.28% | 80.58% | -0.30% |
| **uterus** | 33,444,002 | 33,444,002 |  | 24,005,578 | 23,129,484 |  | 71.78% | 69.16% | 2.62% |
| **pancreas** | 30,595,568 | 30,595,568 |  | 12,789,115 | 12,277,691 |  | 41.80% | 40.13% | 1.67% |
| **lung** | 31,533,498 | 31,533,498 |  | 14,459,381 | 14,218,915 |  | 45.85% | 45.09% | 0.76% |
| **skin** | 34,442,464 | 34,442,464 |  | 18,877,527 | 18,709,209 |  | 54.81% | 54.32% | 0.49% |
| **eye** | 33,006,509 | 33,006,509 |  | 25,395,810 | 25,111,320 |  | 76.94% | 76.08% | 0.86% |
| **brisket** | 34,893,064 | 34,893,064 |  | 28,859,725 | 28,331,694 |  | 82.71% | 81.20% | 1.51% |
| **shin skin** | 34,612,367 | 34,612,367 |  | 27,318,226 | 27,176,155 |  | 78.93% | 78.52% | 0.41% |
| **liver** | 33,476,266 | 33,476,266 |  | 21,787,722 | 21,644,194 |  | 65.08% | 64.66% | 0.42% |
| **bone marrow** | 30,975,506 | 30,975,506 |  | 14,834,302 | 14,525,590 |  | 47.89% | 46.89% | 1.00% |
| **fascia** | 33,316,764 | 33,316,764 |  | 24,302,712 | 23,572,203 |  | 72.94% | 70.75% | 2.19% |
| **cerebrum** | 30,887,821 | 30,887,821 |  | 15,993,857 | 15,667,693 |  | 51.78% | 50.72% | 1.06% |
| **gizzard** | 31,537,118 | 31,537,118 |  | 17,890,098 | 17,613,831 |  | 56.73% | 55.85% | 0.88% |
| **immatured egg** | 32,009,437 | 32,009,437 |  | 20,798,084 | 20,241,417 |  | 64.97% | 63.24% | 1.73% |
| **cockscomb** | 31,936,332 | 31,936,332 |  | 20,702,519 | 20,177,111 |  | 64.82% | 63.18% | 1.64% |
| **spleen** | 36,125,957 | 36,125,957 |  | 26,046,892 | 25,265,226 |  | 72.10% | 69.94% | 2.16% |

**Table S2 Brief statistic results of DNA methylation calling by using Bismark**

|  | **Reads mapping** | | | |  | **DNA Methyl-sites calling** | | | | | | | | | |
| --- | --- | --- | --- | --- | --- | --- | --- | --- | --- | --- | --- | --- | --- | --- | --- |
|  | **# of input read pairs** | **uniquely mapped read pairs** | **duplicately mapped read pairs** | **% Aligned** |  |  | **# of methylated sites** | | | |  | **# of unmethylated sites** | | | |
|  |  |  |  |  |  | **total number of C** | **CpG** | **CHG** | **CHH** | **unknown** |  | **CpG** | **CHG** | **CHH** | **unknown** |
| **matured egg** | 6,246,607 | 3,202,710 | 146,668 | 51.30% |  | 177,424,550 | 10,090,764 | 108,715 | 168,950 | 69,925 |  | 22,972,425 | 49,596,398 | 94,487,298 | 69,925 |
| **cerebellum** | 6,291,610 | 3,189,875 | 149,156 | 50.70% |  | 176,214,564 | 11,154,650 | 287,582 | 730,298 | 70,425 |  | 21,995,666 | 48,678,410 | 93,367,958 | 70,425 |
| **gallbladder** | 5,738,180 | 3,013,478 | 121,766 | 52.50% |  | 158,842,095 | 9,906,046 | 108,148 | 184,254 | 60,739 |  | 16,668,585 | 44,823,958 | 87,151,104 | 60,739 |
| **kidney** | 5,470,502 | 2,775,475 | 124,653 | 50.70% |  | 152,054,891 | 9,266,333 | 101,140 | 163,818 | 59,102 |  | 18,100,440 | 43,135,610 | 81,287,550 | 59,102 |
| **heart** | 5,462,739 | 2,824,787 | 124,043 | 51.70% |  | 153,050,760 | 9,502,121 | 109,858 | 188,542 | 56,357 |  | 18,413,467 | 43,055,170 | 81,781,602 | 56,357 |
| **uterus** | 6,046,764 | 3,010,398 | 137,903 | 49.80% |  | 164,719,944 | 10,207,930 | 116,814 | 183,400 | 64,447 |  | 18,658,769 | 46,895,616 | 88,657,415 | 64,447 |
| **pancreas** | 7,100,215 | 3,689,936 | 145,578 | 52.00% |  | 192,091,263 | 10,633,093 | 115,692 | 198,329 | 76,909 |  | 21,194,789 | 54,711,606 | 105,237,754 | 76,909 |
| **lung** | 5,640,120 | 2,814,000 | 128,373 | 49.90% |  | 156,476,456 | 10,726,832 | 112,441 | 168,610 | 61,561 |  | 17,672,022 | 44,217,572 | 83,578,979 | 61,561 |
| **skin** | 7,226,309 | 3,939,683 | 135,998 | 54.50% |  | 209,619,973 | 12,248,531 | 142,113 | 234,731 | 88,986 |  | 20,886,000 | 57,985,048 | 118,123,550 | 88,986 |
| **eye** | 6,956,141 | 3,724,270 | 156,398 | 53.50% |  | 204,176,024 | 11,928,449 | 149,862 | 290,114 | 82,685 |  | 26,886,495 | 56,866,119 | 108,054,985 | 82,685 |
| **brisket** | 6,042,106 | 3,075,504 | 137,846 | 50.90% |  | 168,447,005 | 10,221,292 | 115,284 | 191,098 | 62,926 |  | 19,895,840 | 47,359,127 | 90,664,364 | 62,926 |
| **shin skin** | 5,924,463 | 3,290,884 | 103,509 | 55.50% |  | 163,206,357 | 8,611,448 | 91,870 | 178,921 | 64,236 |  | 13,727,727 | 45,270,794 | 95,325,597 | 64,236 |
| **liver** | 6,744,208 | 3,709,338 | 58,791 | 55.00% |  | 178,373,598 | 5,066,885 | 70,141 | 169,883 | 93,935 |  | 20,592,744 | 47,784,049 | 104,689,896 | 93,935 |
| **bone marrow** | 5,736,011 | 3,101,124 | 122,834 | 54.10% |  | 171,958,290 | 11,946,629 | 126,285 | 193,306 | 68,689 |  | 17,351,677 | 47,916,458 | 94,423,935 | 68,689 |
| **fascia** | 5,720,194 | 2,936,675 | 141,560 | 51.30% |  | 162,946,393 | 9,777,589 | 104,240 | 171,629 | 61,930 |  | 19,511,561 | 44,900,158 | 88,481,216 | 61,930 |
| **cerebrum** | 6,078,989 | 3,164,808 | 138,464 | 52.10% |  | 175,844,788 | 11,622,932 | 398,627 | 1,051,592 | 69,578 |  | 20,123,525 | 48,954,151 | 93,693,961 | 69,578 |
| **gizzard** | 5,731,878 | 2,919,286 | 139,198 | 50.90% |  | 157,680,918 | 9,287,318 | 114,491 | 191,388 | 61,553 |  | 17,566,145 | 45,089,221 | 85,432,355 | 61,553 |
| **immatured egg** | 6,741,258 | 3,376,822 | 157,829 | 50.10% |  | 184,333,531 | 11,639,636 | 127,296 | 199,814 | 69,369 |  | 21,949,289 | 52,673,025 | 97,744,471 | 69,369 |
| **cockscomb** | 5,948,687 | 3,221,231 | 120,828 | 54.20% |  | 167,236,051 | 10,036,142 | 114,617 | 194,618 | 68,464 |  | 15,448,653 | 47,286,541 | 94,155,480 | 68,464 |
| **spleen** | 6,307,517 | 3,697,919 | 48,478 | 58.60% |  | 168,693,342 | 5,252,124 | 71,465 | 183,606 | 95,076 |  | 12,725,419 | 44,052,784 | 106,407,944 | 95,076 |

**Table S3 Coverage of CpG site using bismark**

|  | **CpG sites covered reads** | | |  | **over 10 reads covered sites** | |
| --- | --- | --- | --- | --- | --- | --- |
|  | **covered read**  **counts** | **covered sites** | **average**  **depth** |  | **# of sites** | **% coverage** |
| **matured egg** | 9,675,068 | 643,124 | 15.0439 |  | 199,960 | 31.09% |
| **cerebellum** | 9,663,846 | 561,945 | 17.1971 |  | 186,121 | 33.12% |
| **gallbladder** | 7,716,427 | 833,674 | 9.25593 |  | 157,716 | 18.92% |
| **kidney** | 7,999,973 | 561,329 | 14.2518 |  | 154,486 | 27.52% |
| **heart** | 8,090,597 | 558,430 | 14.4881 |  | 175,955 | 31.51% |
| **uterus** | 8,429,812 | 539,925 | 15.6129 |  | 148,693 | 27.54% |
| **pancreas** | 9,421,148 | 971,844 | 9.69409 |  | 175,665 | 18.08% |
| **lung** | 8,340,439 | 481,279 | 17.3297 |  | 151,143 | 31.40% |
| **skin** | 9,442,747 | 786,485 | 12.0063 |  | 140,994 | 17.93% |
| **eye** | 11,776,182 | 696,471 | 16.9084 |  | 183,366 | 26.33% |
| **brisket** | 8,718,717 | 517,608 | 16.8442 |  | 173,457 | 33.51% |
| **shin skin** | 6,302,115 | 1,154,300 | 5.45969 |  | 142,064 | 12.31% |
| **liver** | 7,715,251 | 1,762,606 | 4.37718 |  | 169,345 | 9.61% |
| **bone marrow** | 8,305,096 | 545,557 | 15.2231 |  | 161,598 | 29.62% |
| **fascia** | 8,160,086 | 579,453 | 14.0824 |  | 205,273 | 35.43% |
| **cerebrum** | 9,246,448 | 569,705 | 16.2302 |  | 189,787 | 33.31% |
| **gizzard** | 7,628,357 | 614,383 | 12.4163 |  | 130,760 | 21.28% |
| **immatured egg** | 9,668,805 | 614,414 | 15.7366 |  | 212,965 | 34.66% |
| **cockscomb** | 7,071,690 | 884,061 | 7.9991 |  | 119,845 | 13.56% |
| **spleen** | 4,992,523 | 1,652,532 | 3.02114 |  | 83,937 | 5.08% |

**Table S4 Correlation matrix from transcriptome among the twenty tissues**

| **Tissues** |  | **brisket** | **liver** | **bone marrow** | **fascia** | **cerebrum** | **gizzard** | **immatured egg** | **cockscomb** | **spleen** | **matured egg** | **cerebellum** | **gallbladder** | **kidney** | **heart** | **uterus** | **pancreas** | **lung** | **skin** | **eye** | **shin skin** |
| --- | --- | --- | --- | --- | --- | --- | --- | --- | --- | --- | --- | --- | --- | --- | --- | --- | --- | --- | --- | --- | --- |
|  |  |  |  |  |  |  |  |  |  |  |  |  |  |  |  |  |  |  |  |  |  |
| **brisket** |  | 1 | 0.193 | 0.309 | 0.814 | 0.218 | 0.355 | 0.328 | 0.307 | 0.352 | 0.304 | 0.166 | 0.313 | 0.214 | 0.554 | 0.194 | 0.152 | 0.334 | 0.474 | 0.426 | 0.3 |
| **liver** |  | 0.1926107 | 1 | 0.271 | 0.256 | 0.088 | 0.379 | 0.26 | 0.278 | 0.325 | 0.265 | 0.062 | 0.426 | 0.471 | 0.195 | 0.231 | 0.272 | 0.28 | 0.263 | 0.061 | 0.255 |
| **bone marrow** |  | 0.309151 | 0.271 | 1 | 0.351 | 0.148 | 0.394 | 0.448 | 0.375 | 0.574 | 0.341 | 0.162 | 0.447 | 0.291 | 0.334 | 0.27 | 0.223 | 0.567 | 0.431 | 0.311 | 0.35 |
| **fascia** |  | 0.8139473 | 0.256 | 0.351 | 1 | 0.212 | 0.472 | 0.399 | 0.409 | 0.45 | 0.377 | 0.187 | 0.407 | 0.275 | 0.557 | 0.277 | 0.274 | 0.369 | 0.575 | 0.438 | 0.376 |
| **cerebrum** |  | 0.2178542 | 0.088 | 0.148 | 0.212 | 1 | 0.255 | 0.23 | 0.187 | 0.188 | 0.192 | 0.819 | 0.217 | 0.094 | 0.219 | 0.119 | 0.124 | 0.269 | 0.227 | 0.278 | 0.171 |
| **gizzard** |  | 0.3545296 | 0.379 | 0.394 | 0.472 | 0.255 | 1 | 0.428 | 0.406 | 0.442 | 0.385 | 0.22 | 0.513 | 0.365 | 0.387 | 0.356 | 0.337 | 0.493 | 0.489 | 0.297 | 0.369 |
| **immatured egg** |  | 0.3276428 | 0.26 | 0.448 | 0.399 | 0.23 | 0.428 | 1 | 0.384 | 0.456 | 0.638 | 0.235 | 0.429 | 0.269 | 0.378 | 0.337 | 0.296 | 0.472 | 0.473 | 0.29 | 0.385 |
| **cockscomb** |  | 0.3066604 | 0.278 | 0.375 | 0.409 | 0.187 | 0.406 | 0.384 | 1 | 0.445 | 0.453 | 0.278 | 0.465 | 0.313 | 0.33 | 0.254 | 0.24 | 0.432 | 0.629 | 0.23 | 0.613 |
| **spleen** |  | 0.3520442 | 0.325 | 0.574 | 0.45 | 0.188 | 0.442 | 0.456 | 0.445 | 1 | 0.46 | 0.208 | 0.464 | 0.343 | 0.39 | 0.37 | 0.273 | 0.531 | 0.543 | 0.237 | 0.437 |
| **matured egg** |  | 0.3036865 | 0.265 | 0.341 | 0.377 | 0.192 | 0.385 | 0.638 | 0.453 | 0.46 | 1 | 0.229 | 0.47 | 0.263 | 0.368 | 0.297 | 0.232 | 0.469 | 0.451 | 0.223 | 0.393 |
| **cerebellum** |  | 0.1660998 | 0.062 | 0.162 | 0.187 | 0.819 | 0.22 | 0.235 | 0.278 | 0.208 | 0.229 | 1 | 0.206 | 0.116 | 0.212 | 0.14 | 0.112 | 0.256 | 0.247 | 0.256 | 0.214 |
| **gallbladder** |  | 0.3125633 | 0.426 | 0.447 | 0.407 | 0.217 | 0.513 | 0.429 | 0.465 | 0.464 | 0.47 | 0.206 | 1 | 0.462 | 0.347 | 0.371 | 0.331 | 0.567 | 0.504 | 0.214 | 0.374 |
| **kidney** |  | 0.2135884 | 0.471 | 0.291 | 0.275 | 0.094 | 0.365 | 0.269 | 0.313 | 0.343 | 0.263 | 0.116 | 0.462 | 1 | 0.272 | 0.294 | 0.299 | 0.367 | 0.338 | 0.105 | 0.284 |
| **heart** |  | 0.5543102 | 0.195 | 0.334 | 0.557 | 0.219 | 0.387 | 0.378 | 0.33 | 0.39 | 0.368 | 0.212 | 0.347 | 0.272 | 1 | 0.219 | 0.257 | 0.394 | 0.531 | 0.409 | 0.314 |
| **uterus** |  | 0.1941629 | 0.231 | 0.27 | 0.277 | 0.119 | 0.356 | 0.337 | 0.254 | 0.37 | 0.297 | 0.14 | 0.371 | 0.294 | 0.219 | 1 | 0.286 | 0.39 | 0.364 | 0.205 | 0.305 |
| **pancreas** |  | 0.1518205 | 0.272 | 0.223 | 0.274 | 0.124 | 0.337 | 0.296 | 0.24 | 0.273 | 0.232 | 0.112 | 0.331 | 0.299 | 0.257 | 0.286 | 1 | 0.292 | 0.283 | 0.246 | 0.248 |
| **lung** |  | 0.3342512 | 0.28 | 0.567 | 0.369 | 0.269 | 0.493 | 0.472 | 0.432 | 0.531 | 0.469 | 0.256 | 0.567 | 0.367 | 0.394 | 0.39 | 0.292 | 1 | 0.511 | 0.325 | 0.39 |
| **skin** |  | 0.4744756 | 0.263 | 0.431 | 0.575 | 0.227 | 0.489 | 0.473 | 0.629 | 0.543 | 0.451 | 0.247 | 0.504 | 0.338 | 0.531 | 0.364 | 0.283 | 0.511 | 1 | 0.402 | 0.635 |
| **eye** |  | 0.4262754 | 0.061 | 0.311 | 0.438 | 0.278 | 0.297 | 0.29 | 0.23 | 0.237 | 0.223 | 0.256 | 0.214 | 0.105 | 0.409 | 0.205 | 0.246 | 0.325 | 0.402 | 1 | 0.288 |
| **shin skin** |  | 0.3002092 | 0.255 | 0.35 | 0.376 | 0.171 | 0.369 | 0.385 | 0.613 | 0.437 | 0.393 | 0.214 | 0.374 | 0.284 | 0.314 | 0.305 | 0.248 | 0.39 | 0.635 | 0.288 | 1 |

**Table S5 Enrichment terms of unsupervised k-means clustering**

| **cluster number** |  | **Name** | **# of Entities** | **Overlap** | **Percent Overlap(%)** | **Overlapping Entities** | **p-value** | **Hit type** |
| --- | --- | --- | --- | --- | --- | --- | --- | --- |
| cluster1 |  | sarcomere | 47 | 8 | 17 | MYLK2;ACTN2;KLHL40;OBSCN;MYL1;CKMT2;ABRA;MYOM1 | 1.13E-10 | cellular_component |
|  |  | muscle contraction | 114 | 10 | 8 | ACTN2;MYOM2;CKMT2;MYOM1;MYLK2;MYH13;CLCN1;KCNJ12;TNNT3;MYL1 | 5.30E-10 | biological_process |
|  |  | myofibril | 47 | 7 | 14 | MYH13;OBSCN;ANKRD1;MYL1;NRAP;ABRA;MYOD1 | 4.60E-09 | cellular_component |
| cluster2 |  | myofibril | 47 | 8 | 17 | MYL2;MYBPC3;TMOD1;LRRC10;MYH7;CAPN3;MYH15;TNNT2 | 6.54E-12 | cellular_component |
|  |  | muscle filament sliding | 39 | 7 | 17 | MYL2;MYBPC3;TMOD1;TNNC1;MYH7;TNNI1;TNNT2 | 9.69E-11 | biological_process |
|  |  | cardiac muscle contraction | 51 | 7 | 13 | MYL2;CSRP3;MYBPC3;TNNC1;TNNT2;NKX2-5;SCN5A | 7.04E-10 | biological_process |
| cluster3 |  | cellular response to gonadotropin stimulus | 19 | 6 | 31 | STAR;HSD3B3;CYP17A1;CYP11A1;PGR;LHCGR | 5.67E-11 | biological_process |
|  |  | structural constituent of muscle | 52 | 7 | 13 | MYL2;MYL3;PDLIM3;TPM1;NEXN;TPM4;MYH11 | 8.78E-10 | molecular_function |
|  |  | testosteron biosynthetic process | 6 | 4 | 66 | STAR;HSD3B2;HSD17B1;CYP11A1 | 2.62E-09 | biological_process |
| cluster4 |  | small molecule metabolic process | 1392 | 55 | 3 | GSTZ1;ACMSD;PEX11A;EPT1;ABCG8;GYS2;CAT;HAAO;HMGCL;PCBD1;SLC25A;ENPP7;DIO1;FABP1;HAL;IYD;AMDHD1;KYNU;MTTP;SLC2A2;HMGCS2;XDH;ADH6;CYP1A1;FAH;NUDT5;AMN;HAO1;SHMT1;HSD3B7;HGD;AGMAT;GATM;AGXT;UPP2;FTCD;APOB;KMO;UROC1;ABCB11;PEMT;CYB5A;ACOX2;GALE;IDO2;FBP1;TCN2;LTC4S;AHCY;PAH;APOA5;ALDOB;HPD;ABCG5;MAT1A | 5.02E-26 | biological_process |
|  |  | cellular nitrogen compound metabolic process | 185 | 24 | 12 | GSTZ1;ACMSD;HAL;IYD;HAO1;AMDHD1;SHMT1;KYNU;IDO2;HGD;HAAO;AGMAT;GATM;AGXT;PCBD1;AHCY;PAH;FTCD;FAH;KMO;HPD;UROC1;MAT1A;DIO1 | 4.18E-24 | biological_process |
|  |  | catalytic activity | 713 | 32 | 4 | GSTZ1;ACMSD;ACSM3;GYS2;ECI2;HMGCL;ENPP7;HAL;KYNU;HMGCS2;XDH;habp2;CYP1A1;FAH;HAO1;SHMT1;SLC3A1;PROZ;AGXT;CFI;UPP2;FTCD;HGFAC;HOGA1;C2;GALE;FBP1;LIPC;PAH;HAO2;MASP2;ALDOB | 1.09E-17 | molecular_function |
| cluster5 |  | sequence-specific DNA binding transcription factor activity | 1135 | 17 | 1 | CDX2;ISL1;NROB2;FOXF1;PDX1;PTF1A;BARX1;MYRFL;SPDEF;HNF1B;NKX6-3;RBPJL;FOXB1;HMX3;TBX4;TSHZ3;NKX2-8;SLC26A3;NR5A2 | 2.25E-07 | molecular_function |
|  |  | pancreas development | 43 | 5 | 11 | ISL1;FOXF1;PDX1;IHH;PTF1A | 9.99E-0.7 | biological_process |
|  |  | endocrine pancreas development | 45 | 5 | 11 | HNF1B;INS;NR5A2;PDX1;PTF1A | 1.26E-06 | biological_process |
| cluster6 |  | synaptic transmission | 452 | 15 | 3 | NEFL;RIT2;HTR7;NPY5R;DRD2;KCNK10;SLC1A3;OPRK1;NPTX2;PDYN;CHRM2;GABRA5;AKAP5;HAP1;KCNG1 | 1.20E-09 | biological_process |
|  |  | neuromodulator | 61 | 7 | 11 | PDYN;AGT;GIP;IAPP;GRP;AVP;CCK | 7.36E-09 | Pathway Studio Ontology |
|  |  | transmembrane transport | 717 | 17 | 2 | SLC26A1;SLC1A3;UNC79;RYR2;SLC3OA2;SLCO4A1;GABRA5;SLC3OA10;ATP2A2;SLCO1B;SLC4A5;SLCO1A2;MFSD4;SLC6A7;ATP7B;AVP;KCNG1 | 1.35E-08 | biological_process |
| cluster7 |  | hemoglobin complex | 15 | 4 | 26 | HBG1;HBE1;HBB;HBZ | 1.25E-07 | cellular_component |
|  |  | cytokine receptor activity | 40 | 5 | 12 | IL21R;CSF3R;IL2RB;IL7R;FLT3 | 1.69E-07 | molecular_function |
|  |  | oxygen transporter activity | 18 | 4 | 22 | HBG1;HBE1;HBB;HBZ | 2.75E-07 | molecular_function |
| cluster8 |  | lipid metabolic process | 495 | 12 | 2 | SRD5A1;ACER1;ELOVL3;TTPA;FADS1;ELOVL4;ELOVL6;OLAH;CIDEA;FADS6;SPTL3;CERS3 | 3.11E-08 | biological_process |
|  |  | fatty acid biosynthesis | 15 | 4 | 26 | ELOVL6;ELOVL4;OLAH;ELOVL3 | 6.78E-08 | Pathway Studio Ontology |
|  |  | fatty acid elongation, stuated fatty acid | 5 | 3 | 60 | ELOVL6;ELOVL4;ELOVL3 | 2.70E-07 | biological_process |
| cluster9 |  | axonemal dynein complex | 16 | 4 | 25 | DNAH1;DNAH5;DNAI2;DNAH10 | 1.34E-07 | cellular_component |
|  |  | dynein complex | 35 | 4 | 11 | DNAH1;DNAH5;DNAI2;DNAH10 | 3.70E-06 | cellular_component |
|  |  | cilium or flagellum-dependent cell motility | 21 | 3 | 14 | DNAH1;ROPN1L;DNAH5 | 3.28E-05 | biological_process |
| cluster10 |  | visual perception | 211 | 37 | 17 | PPEF2;CRYBB2;RS1;VSX2;GLRA1;SLC24A1;SIX6;GUCA1C;PDC;GUCA1A;RDH8;KERA;NR2E3;RRH;C2orf71;VSX1;RBP3;SAG;GABRR1;GUCY2F;IMPG1;MYO3A;CABP4;CNGB3;OPN5;ARR3;CRYBB1;CRYAA;FSCN2;PDE6H;PDE6B;CRX;PRPH2;RPE65;CRYBB3;RGR;GUCA1B | 1.58E-50 | biological_process |
|  |  | phtotransduction, visible light | 89 | 11 | 12 | GUCY2F;CNGB3;SLC24A1;GUCA1C;GUCA1A;RDH8;PDE6B;RPE65;RBP3;GUCA1B;SAG | 1.27E-13 | biological_process |
|  |  | phtotransduction | 34 | 8 | 23 | CABP4;OPN5;PDC;GUCA1A;NR2E3;RRH;RGR;GUCA1B | 1.30E-12 | biological_process |
| cluster11 |  | ion transduction | 650 | 22 | 3 | GRIK2;SLC5A11;SLC5A9;SLC26A6;SLC5A12;GRID2;ATP6V1G3;SLC6A20;SLC12A3;KCNJ1;SLC7A9;SLC12A1;SLC5A10;SLC9A3;SLC6A18;SLC6A19;SLC4A9;CLDN16;CACNA1I;SLC22A7;SLC26A4;SLC34A1 | 2.80E-20 | biological_process |
|  |  | transmembrane transport | 717 | 20 | 2 | SLC5A11;SLC25A48;SLC5A9;RHCG;SLC26A6;SLC5A12;ATP6V1G3;SLC6A20;SLC12A3;SLC7A9;SLC12A1;SLC5A10;SLC9A3;SLC6A18;SLC6A19;SLC4A9;SLC22A7;SLC26A4;SLC34A1;BSND | 1.00E-16 | biological_process |
|  |  | Na+ influx co-transport | 96 | 11 | 11 | SLC5A12;SLC12A1;SLC5A10;SLC9A3;SLC5A11;SLC6A20;SLC12A3;SLC6A19;SLC4A9;SLC5A9;SLC34A1 | 9.45E-16 | Pathway Studio Ontology |
| cluster12 |  | synapse | 422 | 35 | 8 | GRIK2;GABRA1;LRRC7;SLC17A6;SLC32A1;SNAP26;GSG1L;GAP43;GABRG2;PHACTR1;SYN3;SNCA;CADM2;KCTD16;GRIN2B;SEPT3;TRIM9;GRIN1;SV2C;CNTN2;RIMS3;GABRB3;NETO1;GLRB;GAD2;CPLX2;CPLX3;SYN2;GRIK3;DLGAP2;CPLX;GRIA4;CALB1;BCAN;GLRA3 | 7.83E-31 | cellular_component |
|  |  | synaptic transmission | 452 | 32 | 7 | GRIK2;GABRA1;SLC32A1;SNAP25;SLC5A11;GRM1;GABRG2;KCNAB1;HTR2C;SNCA;GRIN2B;GRIN1;SV2C;PLP1;GABRB3;GLRB;GAD2;DRD5;GRM3;SYN2;GRM5;GRIK3;CACNA1B;CACNA1E;CACNG3;KCNJ6;CPLX1;ADCY8;GRIA4;TAC1;GRM4;GLRA3 | 5.25E-26 | biological_process |
|  |  | cell junction | 664 | 30 | 4 | GRIK2;GABRA1;LRRC7;SLC17A6;SNAP25;GSG1L;GAP43;GABRG2;PHACTR1;SYN3;SNCA;CADM2;KCTD16;GRIN2B;SEPT3;TRIM9;GRIN1;SV2C;RIMS3;GABRB3;NETO1;GLRB;GAD2;CPLX2;CPLX3;SYN2;GRIK3;DLGAP2;GRIA4;GLRA3 | 8.00E-19 | cellular_component |

**Table S6 Enrichment terms of DEGs from clustered tissues**

| **sample names** |  | **Name** | **# of Entities** | **Overlap** | **Percent Overlap(%)** | **Overlapping Entities** | **p-value** | **Hit type** |
| --- | --- | --- | --- | --- | --- | --- | --- | --- |
| bone marrow vs spleen |  | extracellular matrix organaization | 290 | 11 | 3 | COL12A;ITGB8;COL21A1;RXFP1;NRXN1;FBLN5;DST;DMD;ABI3BP;NPNT;COL14A1 | 2.57E-08 | biological_process |
|  |  | positive regulation of synaptic transmission, glutamatergic | 22 | 4 | 18 | EGFR;NTRK2;NLGN1;NRXN1 | 1.72E-06 | biological_process |
|  |  | presynaptic membrane assembly | 9 | 3 | 33 | NLGN1;NRXN1;PTPRD | 5.24E-06 | biological_process |
| brisket vs heart |  | structural constituent of muscle | 52 | 9 | 17 | MYL3;MYBPC3;JPH1;MYL2;MYBPH;TPM1;TPM4;MYL1;SORBS2 | 1.69E-10 | molecular_function |
|  |  | muscle filament sliding | 39 | 8 | 20 | MYL3;MYBPC3;MYL2;TPM1;ACTA1;TPM4;TNNT3;MYL1 | 4.27E-10 | biological_process |
|  |  | sarcomere | 47 | 8 | 17 | MYL3;MYBPC3;MYL2;TPM1;ACTA1;RYR2;MYL1;MEF2C | 2.07E-09 | cellular_component |
| eye vs cerebellum |  | proteinaceous extracellular matrix | 318 | 19 | 5 | PODN;ECM2;LTBP2;MATN2;DMBT1;COL1A2;THBS4;COCH;COL3A1;MMP7;SMOC2;CCDC80;WNT10A;WNT3A;MATN1;COL14A1;LAMA3;IMPG2;COL4A4 | 5.62E-12 | cellular_component |
|  |  | extracellular matrix organaization | 290 | 17 | 5 | ECM2;COL4A5;NRXN1;COL1A2;COL3A1;MMP7;SMOC2;MUSK;CCDC80;RXFP1;ABI3BP;WNT3A;MATN1;COL14A1;COL6A3;LAMA3;COL4A4 | 1.04E-10 | biological_process |
|  |  | extracellular matrix polymerization | 97 | 10 | 10 | ECM2;COL4A5;MATN2;COL1A2;THBS4;MATN1;COL3A1;COL14A1;COL6SA3;COL4A4 | 3.26E-09 | Pathway Studio Ontology |
| eye vs cerebrum |  | proteinaceous extracellular matrix | 318 | 16 | 5 | PODN;ECM2;SPARCL1;LTBP2;MATN2;DMBT1;COCH;MMP7;SMOC2;CPA6;CCDC80;WNT10A;WNT3A;COL14A1;IMPG2;COL4A4 | 1.05E-08 | cellular_component |
|  |  | transmembrane receptor protein tyrosine kinase signaling pathway | 99 | 9 | 9 | MUSK;EPHA7;NTRK2;EPHA6;ADCYAP1R1;SHC3;EPHA5;NRG1;CNKSR1 | 1.37E-07 | biological_process |
|  |  | sarcomere organization | 33 | 6 | 18 | MYBPC3;FHOD3;OBSCN;XIRP1;LDB3;TNNT2 | 2.73E-07 | biological_process |
| fascia vs brisket |  | extracellular matrix polymerization | 97 | 5 | 5 | COL12A1;COL1A2;SPON2;MATN2;COL6A3 | 4.21E-07 | Pathway Studio Ontology |
|  |  | collagen | 98 | 4 | 4 | COL12A1;COL1A2;EMID1;COL6A3 | 2.03E-05 | cellular_component |
|  |  | sarcolemma | 107 | 4 | 3 | DMD;COL6A3;DTNA;SGCZ | 2.87E-05 | cellular_component |
| fascia vs heart |  | muscle filament sliding | 39 | 7 | 17 | MYBPC3;TPM1;ACTA1;TPM4;TNNT3;MYL1;TNNT2 | 5.62E-10 | biological_process |
|  |  | sarcomere | 47 | 7 | 14 | MYBPC3;TPM1;LRRC10;ACTA1;RYR2;MYL1;TNNT2 | 2.23E-09 | cellular_component |
|  |  | muscle contraction | 114 | 8 | 7 | MYBPC3;TPM1;ACTA1;TPM4;CHRND;TNNT3;MYL1;TNNT2 | 6.64E-08 | biological_process |
| kidney vs liver |  | synaptic transmission | 452 | 51 | 11 | HCN1;GRIK2;CHRNA4;RIMS1;GABRG2;CHAT;KCNV2;DTNA;CDH8;GABRA2;KCNK3;CHRM2;CHRM3;GRM3;SLC1A2;KCNC1;UNC13C;KCND3;GRIA4;SYT1;GRM4;PANX2;EPB41L1;DLG2;KCNJ11;GABRG1;KCNQ3;KCNC4;SLC5A7;GRM8;KCNA4;GABRA6;CACNB2;KCNH5;KCNQ2;GRIN2B;GJD2;CHRND;SV2C;KCNC2;RASGRF1;GAD2;AMPH;GNGT2;KCNQ4;CLSTN2;CACNG3;UNC13A;FGF14;KCNF1;KCNA1 | 6.29E-22 | biological_process |
|  |  | synapse | 422 | 46 | 10 | GRIK2;OLFM1;NMNAT2;CHRNA4;SYNPR;RIMS1;GABRG2;DTNA;CADM2;CADPS;NLGN1;GABRA2;NETO1;SYT11;CHRM2;CHRM3;LRRC4C;CBLN4;SLC1A2;NRCAM;GLRA2;UNC13C;DMD;LIN7A;GRIA4;SYT1;DLG2;GABRG1;ZNRF2;SPARCL1;GABRA6;SYN3;VWC2L;MAGI2;GLRA4;SYT7;NBEA;BRIN2B;CHRND;SV2C;SYT9;GAD2;AMPH;SYT10;RPH3A;UNC13A | 2.89E-19 | cellular_component |
|  |  | ion transport | 650 | 56 | 8 | HCN1;GRIK2;SLC38A4;CHRNA4;SLC24A3;GABRG2;KCNV2;CNGA1;SLC24A2;TRPM3;SLC6A15;TRPM1;RYR2;CACNA2D1;GABRA2;KCNK3;SLCO3A1;SLC1A2;KCNC1;SLC4A11;GLRA2;KCND3;GRIA4;PANX2;SLC7A10;KCNJ11;GABRG1;KCNQ3;KCNIP4;KCNC4;SLC5A7;SLC3A1;SLCO1B3;KCNA4;GABRA6;CACNB2;KCNH5;ATP7A;KCNQ2;ATP2B2;AQP9;GLRA4;ANO1;GRIN2B;CHRND;SLCO1A2;LRRC38;KCNC2;SLCO1C1;KCNQ4;CACNG3;TRPC7;SCN3A;KCNF1;SLC4A10;KCNA1 | 2.04E-18 | biological_process |
| matured egg vs immatured egg |  | regulation of mitosis | 22 | 2 | 9 | KIF20B;BORA | 1.32E-04 | biological_process |
|  |  | STC | 1 | 1 | 100 | STC1 | 6.08E-04 | Pathway Studio Ontology |
|  |  | positive regulation of histone H4K20 methylation | 1 | 1 | 100 | BRCA1 | 7.82E-04 | biological_process |
| shin skin vs scockscomb |  | extracellular matrix | 250 | 5 | 1 | SMOC2;ALPL;ABI3BP;ACAN;ADAMTSL2 | 1.07E-05 | cellular_component |
|  |  | D1 dopamine receptor binding | 11 | 2 | 18 | AGTR1;PPP1R1B | 7.41E-05 | molecular_function |
|  |  | interstitial matrix | 20 | 2 | 10 | SMOC2;ABI3BP | 2.58E-04 | cellular_component |
| skin vs shin skin |  | M band | 21 | 4 | 19 | OBSCN;CMYA4;SMPX;MYOM1 | 1.66E-08 | cellular_component |
|  |  | striated muscle myosin thick filament | 4 | 2 | 50 | OBSCN;MYOM1 | 1.07E-05 | cellular_component |
|  |  | sarcomere | 47 | 3 | 6 | OBSCN;MYLK2;MYOM1 | 3.55E-05 | cellular_component |

**Table S7 Number of alternative splicing events by filtering critera.**

| **Splicing events** | **Alternative 3‘ exon** | **Alternative 5‘ exon** | **Mutually Exclusive Exon** | **Retained intron** | **cassette exon** |
| --- | --- | --- | --- | --- | --- |
| **total observations^a^** | 6,859 | 4,188 | 2,303 | 9,213 | 22,482 |
| **canonical splice sites filtering (GT-AG, CT-AC)^b^** | 5,541 | 3,237 | 1,919 | 6,720 | 21,688 |
| **Calculable exons in total tissues^c^** | 2,471 | 1,516 | 471 | 3,460 | 7,338 |
| **Read depth filtering^d^** | 669 | 479 | NA | 1,107 | 2,910 |

^a^ The number of sites that appeared to be pair-wise comparison using rMATS (FDR <= 0.05)

^b^ Criteria that the exon splice site and complementary sequence of thes were canonical splice sites.

^C^ Excluding sites that were not calculated due to lack of depth

^d^ Evidence reads(reads split or paired reads) depth were insufficient.

**Table S8 The number of DEUs in developmental stage.**

|  |  |  |  |  |
| --- | --- | --- | --- | --- |
| **Type** |  | **Computable exons^a^** | **Alternatively Used Exons^b^** |  |
| exon skipping |  | 6,726 | 82 |  |
| alternative 3' exon |  | 3,117 | 29 |  |
| alternative 5' exon |  | 1,908 | 29 |  |

^a^DNA methylated sites that pass the read depth filtering criteria.

^b^FDR <= 0.05

**Table S9 Enrichment terms of developmental stage DEUs**

| Enrichment Score: 3.1835226982388805 | |
| --- | --- |
| REFSEQ_MRNA^a^ | Gene Name |
| NM_001031380 | poly(U) binding splicing factor 60KDa(PUF60) |
| NM_001001305 | serine/arginine-rich splicing factor 2(SRSF2) |
| NM_204678 | transformer 2 beta homolog (Drosophila)(TRA2B) |
| NM_001080861 | eukaryotic translation initiation factor 4H(EIF4H) |
| NM_001031296 | serine/arginine-rich splicing factor 11(SRSF11) |
| NM_001252015 | chromosome 2 C6orf52 homolog(C2H6orf52) |
| NM_001195554 | serine/arginine-rich splicing factor 3(SRSF3) |

^a^ Ensembl Gene Names were converted to refSeq Names

**Table S10 GSEA results of 160 switch-like exons**

| **Name** | **# of Entities** | **Overlap** | **Percent**  **Overlap(%)** | **Overlapping Entities** | **p-value** |
| --- | --- | --- | --- | --- | --- |
| positive regulation of mitotic metaphase-anaphase transition | 8 | 2 | 25 | NSMCE2;CENPE | 6.63386E-05 |
| histone H3-K36 demethylation | 8 | 2 | 25 | KDM4B;KDM2B | 6.63386E-05 |
| poly(A) RNA binding | 1249 | 9 | 0 | DDX17;SORBS2;LSM14B;C4BPA;SRSF3;TRA2B;EIF4ENIF1;FLNB;EIF4H | 0.000102843 |
| npBAF complex | 12 | 2 | 16 | SMARCA2;SS18 | 0.000155753 |
| nucleus | 6977 | 22 | 0 | ALOX5;CENPE;SPECC1;MED12L;FGFR1;LRMP;PPP2R5D;SS18;TAF2;NSMCE2;SRSF3;TRA2B;SMARCA2;PICALM;SORBS2;DDX17;SLU7;SORBS1;KDM4B;FBXO25;EIF4ENIF1;KDM2B | 0.000161295 |
| cytosol | 3691 | 15 | 0 | ALOX5;SORBS1;CENPE;SEC31A;FGFR1;FLNB;VPS29;PPM1B;PPP2R5D;EIF4ENIF1;EIF4H;TPM3;PIP5K1B;URM1;FAM126A | 0.000244484 |
| stress fiber | 78 | 3 | 3 | FLNB;TPM3;SORBS1 | 0.000247849 |
| dioxygenase activity | 83 | 3 | 3 | KDM4B;KDM2B;ALOX5 | 0.000297731 |
| phosphatidylinositol phosphorylation | 97 | 3 | 3 | FGFR1;FAM126A;PIP5K1B | 0.00047061 |
| histone demethylase activity | 23 | 2 | 8 | KDM4B;KDM2B | 0.000590603 |

**Table S11 GSEA of significant negative correlated DNA methylations**

| Name | # of Entities | Overlap | Percent Overlap(%) | Overlapping Entities | p-value |
| --- | --- | --- | --- | --- | --- |
| liver development | 143 | 5 | 3 | NOTCH1;ICMT;PDX1;CITED2;FSCN1 | 3.97E-04 |
| negative regulation of neuron differentiation | 81 | 4 | 4 | NOTCH1;FOXG1;LSM1;MEIS1 | 4.25E-04 |
| D-glucose transmembrane transporter activity | 8 | 2 | 25 | SLC2A10;SLC2A3 | 5.10E-04 |
| pivalyl-CoA mutase activity | 8 | 2 | 25 | DKC1;TRUB2 | 5.10E-04 |
| o-hydroxylaminobenzoate mutase activity | 8 | 2 | 25 | DKC1;TRUB2 | 5.10E-04 |
| lupeol synthase activity | 8 | 2 | 25 | DKC1;TRUB2 | 5.10E-04 |
| baruol synthase activity | 8 | 2 | 25 | DKC1;TRUB2 | 5.10E-04 |
| beta-amyrin synthase activity | 8 | 2 | 25 | DKC1;TRUB2 | 5.10E-04 |
| signal complex assembly | 8 | 2 | 25 | NCK2;PXN | 5.10E-04 |
| cardiac ventricle morphogenesis | 9 | 2 | 22 | NOTCH1;GRHL2 | 6.53E-04 |
| cytosol | 3691 | 29 | 0 | APEH;ATOX1;ABCD2;MAP1LC3C;DOCK1;WIPI2;PALD1;LSM1;PDX1;IRF6;MOV10;GMPPA;NOTCH1;NTRK2;ANAPC5;POLR2E;SNX33;RPLP0;RNF114;PXN;FSCN1;PRDM16;MLST8;SOCS3;NCK2;RILP;SH2B3;ARPC1B;NLRC5 | 7.61E-04 |
| coenzyme A biosynthetic process | 10 | 2 | 20 | COASY;PANK3 | 8.14E-04 |
| cytoplasm | 7242 | 47 | 0 | APEH;ATOX1;KIF1A;B2M;SYTL4;DKC1;DOCK1;PDX1;IRF6;MMP2;MOV10;NOTCH1;NTRK2;NTSR1;MST1;PNN;RASSF3;SNX33;RPLP0;PXN;SLC2A3;FSCN1;TUBB2B;MLX;DND1;ELL;SOCS3;NCK2;ARPC1B;AP5Z1;CITED2;STK25;LURAP1;WIPI2;ABHD14A;THUMPD3;PALD1;LSM1;TRUB2;GMPPA;RNF114;MLST8;PANK3;MOB2;COASY;SLC2A10;NLRC5 | 8.28E-04 |
| regulation of membrane depolarization | 11 | 2 | 18 | NTSR1;B2M | 9.93E-04 |
| cardiac septum morphogenesis | 11 | 2 | 18 | CITED2;NOTCH1 | 9.93E-04 |
| Vitamin B5 (Pantothenate) Metabolism and Biosynthesis of CoA and Holo-ACP | 35 | 2 | 4 | COASY;PANK3 | 1.07E-03 |
| signaling adaptor activity | 12 | 2 | 16 | SH2B3;Sh2b3 | 1.19E-03 |
| prostate gland epithelium morphogenesis | 12 | 2 | 16 | NOTCH1;MMP2 | 1.19E-03 |
| GTP biosynthetic process | 13 | 2 | 15 | NME4;DECR2 | 1.40E-03 |
| UTP biosynthetic process | 13 | 2 | 15 | NME4;DECR2 | 1.40E-03 |
| CTP biosynthetic process | 13 | 2 | 15 | NME4;DECR2 | 1.40E-03 |
| pyrimidine nucleotide metabolic process | 13 | 2 | 15 | NME4;DECR2 | 1.40E-03 |
| isomerase activity | 114 | 4 | 3 | EBP;DKC1;FKBP8;TRUB2 | 1.53E-03 |
| purine nucleotide metabolic process | 14 | 2 | 14 | NME4;DECR2 | 1.63E-03 |
| endocardial cushion development | 14 | 2 | 14 | CITED2;NOTCH1 | 1.63E-03 |
| dorsal-ventral pattern formation | 54 | 3 | 5 | FKBP8;FOXG1;CHRD | 1.66E-03 |
| neural tube development | 55 | 3 | 5 | NOTCH1;FKBP8;GRHL2 | 1.75E-03 |
| SMAD binding | 56 | 3 | 5 | COL1A2;CITED2;PRDM16 | 1.84E-03 |
| pseudouridine synthase activity | 15 | 2 | 13 | DKC1;TRUB2 | 1.87E-03 |
| regulation of Notch signaling pathway | 15 | 2 | 13 | NOTCH1;TSPAN15 | 1.87E-03 |
| left-right axis specification | 15 | 2 | 13 | CITED2;NOTCH1 | 1.87E-03 |
| glucose import | 15 | 2 | 13 | SLC2A10;SLC2A3 | 1.87E-03 |
| nucleoside triphosphate biosynthetic process | 16 | 2 | 12 | NME4;DECR2 | 2.14E-03 |
| RNA modification | 16 | 2 | 12 | DKC1;TRUB2 | 2.14E-03 |
| pseudouridine synthesis | 16 | 2 | 12 | DKC1;TRUB2 | 2.14E-03 |
| cell differentiation | 934 | 11 | 1 | CITED2;ANKS4B;PDX1;IRF6;RNF114;Sh2b3;SH2B3;NOTCH1;FOXG1;NTRK2;PRDM16 | 2.38E-03 |
| glucose transmembrane transporter activity | 17 | 2 | 11 | SLC2A10;SLC2A3 | 2.41E-03 |
| lens morphogenesis in camera-type eye | 17 | 2 | 11 | CITED2;MEIS1 | 2.41E-03 |
| substrate-specific transmembrane transporter activity | 18 | 2 | 11 | SLC2A10;SLC2A3 | 2.71E-03 |
| central nervous system neuron development | 18 | 2 | 11 | FOXG1;NTRK2 | 2.71E-03 |
| negative regulation of anoikis | 18 | 2 | 11 | NOTCH1;NTRK2 | 2.71E-03 |
| in utero embryonic development | 322 | 6 | 1 | ELL;ICMT;CITED2;NOTCH1;GRHL2;RAD51B | 2.78E-03 |
| nucleoside diphosphate phosphorylation | 19 | 2 | 10 | NME4;DECR2 | 3.02E-03 |
| collagen catabolic process | 68 | 3 | 4 | COL1A2;COL11A1;MMP2 | 3.21E-03 |
| cell migration | 231 | 5 | 2 | DOCK1;MMP2;LURAP1;NCK2;FSCN1 | 3.33E-03 |

**Table S12 Three genes crossed when searching for GSMs in the imprintome database**

| **species** | **gene name** | **aliases** | **location** | **status** | **expressed allele** |
| --- | --- | --- | --- | --- | --- |
| *Homo sapiens* | **FOXG1** | BF1,BF2,QIN,FKH2,HBF2,HFK1,HFK2,HFK3,KHL2,FHKL3,FKHL1,FKHL2,FKHL3,FKHL4,HBF-1,HBF-2,HBF-3,FOXG1A,FOXG1B,FOXG1C,HBF-G2 | 14q13 | Predicted | Paternal |
| *Monodelphis domestica* | **MEIS1** |  | AS | Imprinted | Maternal |
| *Homo sapiens* | **PRDM16** | MEL1,PFM13,KIAA1675,MGC166915 | 1p36.23-p33 | Predicted | Paternal |


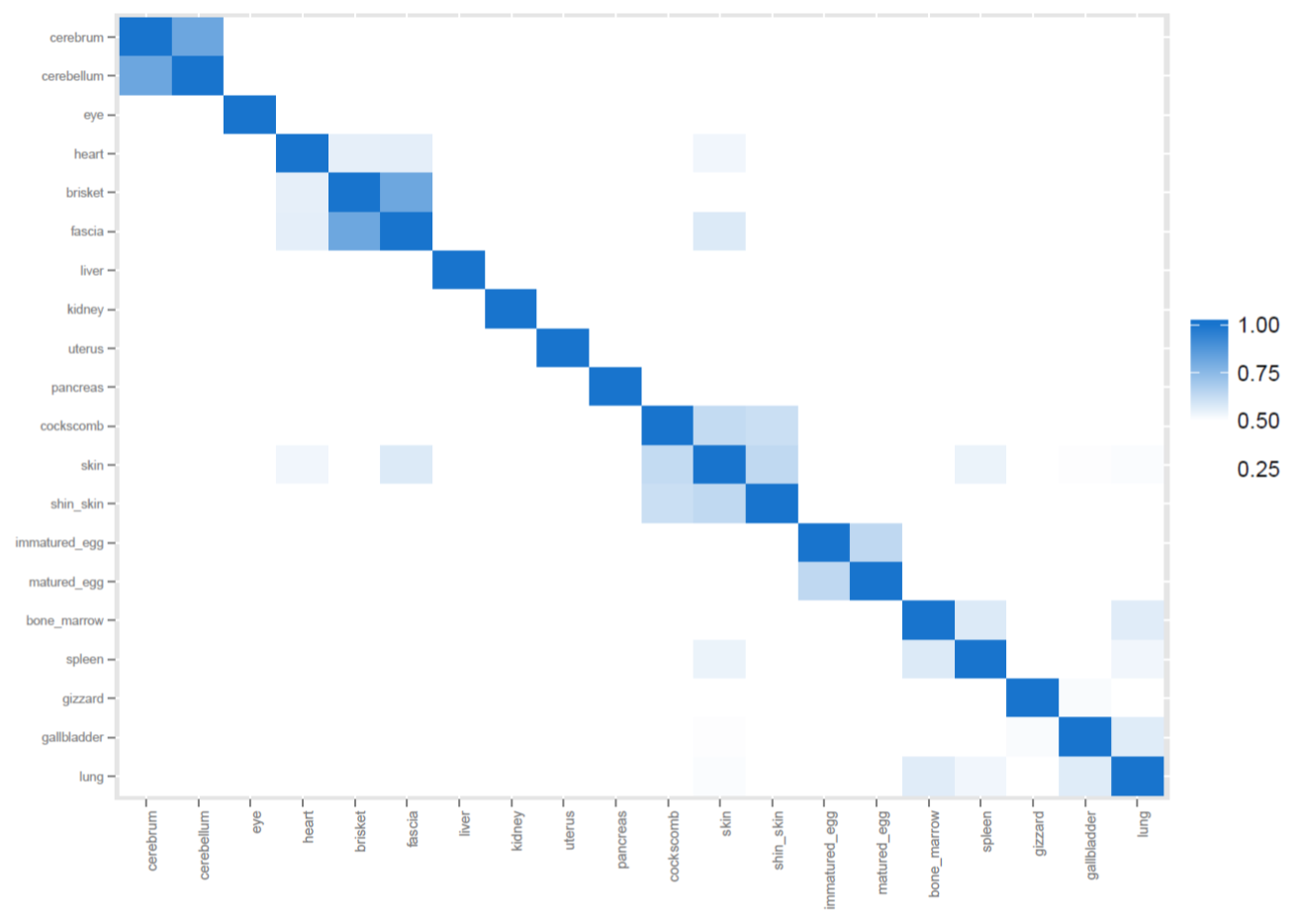


**Figure S1 Correlation plot among the twenty tissues using Gene Expression Values**


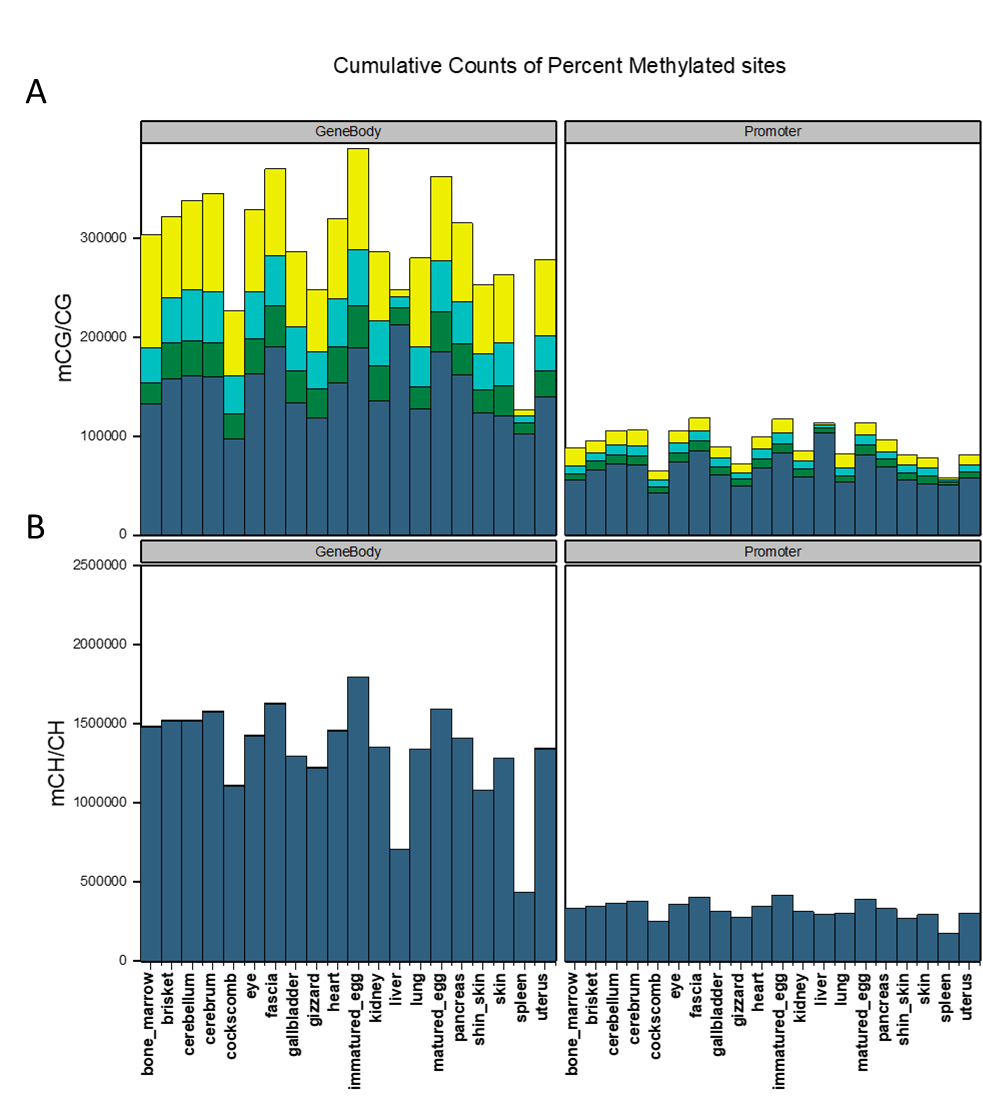


**Figure S2 Cumulative count of DNA methylation Calling results.**

The number of CpG sites(A) and non-CpG sites(B) were counted according to the converted genome information from bismark. The interval was divided and displayed every 0.25(Bright yellow: hypo-methylated DNA; dark blue: hyper-methylated DNA. Cyan: DNA methylation level 0.25–0.5; green: 0.5–0.75).


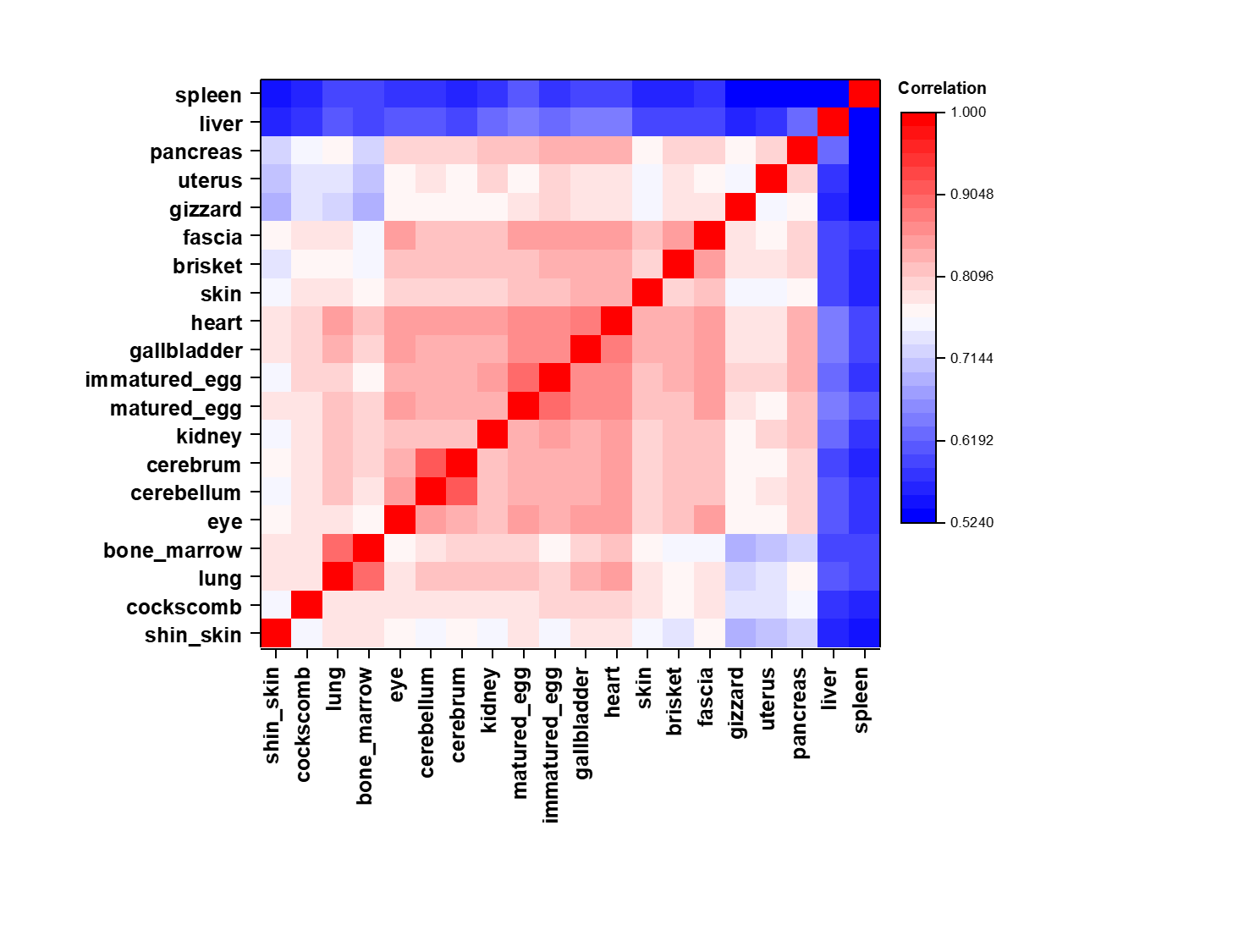


**Figure S3 Correlation plot among the twenty tissues using DNA methylation levels**


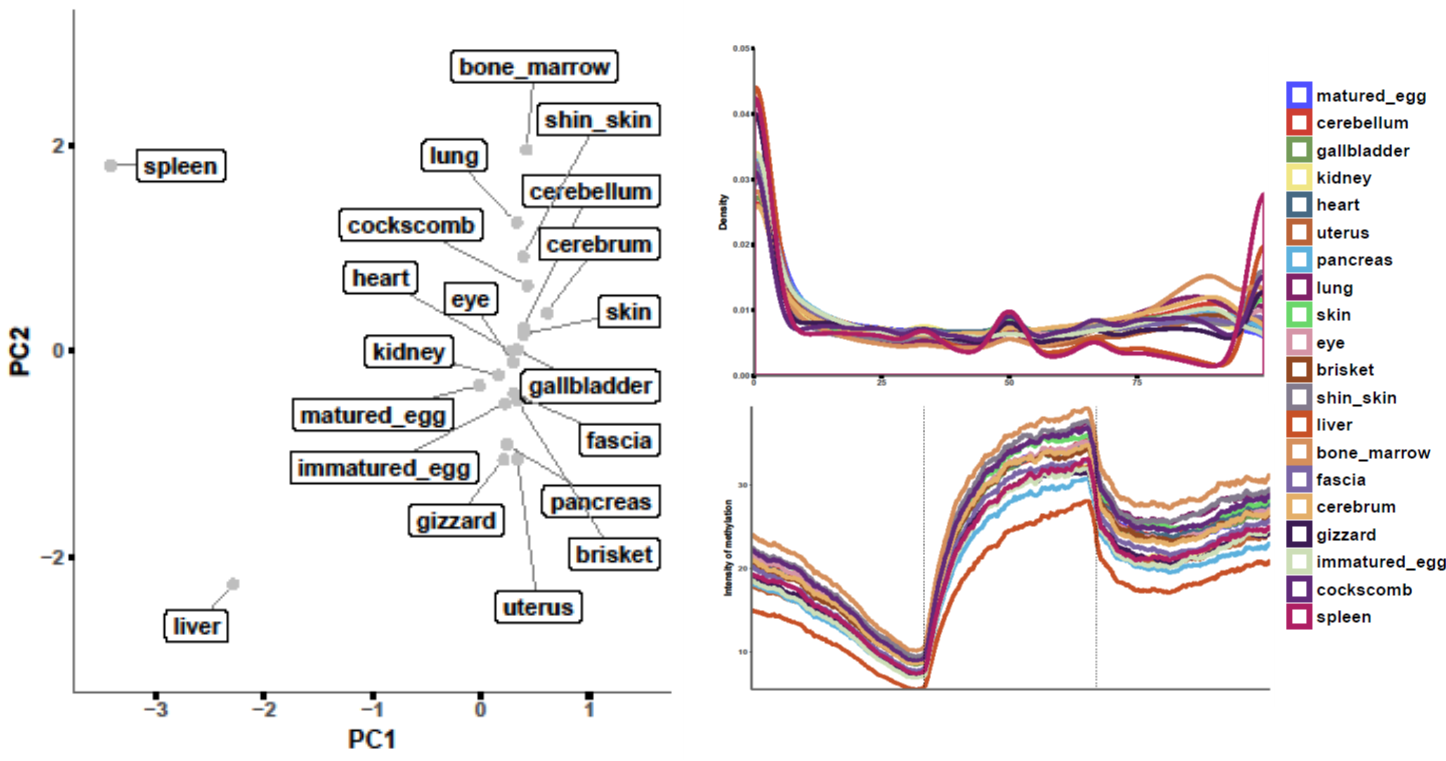


C

B

A

**Figure S4 Overview of DNA methylation levels**

(A) Principal Component Analysis plot of DNA methylation levels. (B) Smoothed and estimated density of DNA methylation in CpG sites. Density estimation was performed by geom_density from ggplot. Each lines represents accumulated intensity from hypo-methylation to hyper-methylation. (C) We assumed the all genes to be same length, and represents an average of DNA-methylation percentages from upstream 2000bp to downstream 2000bp around Transcription Start Site(TSS) and Transcription End Site(TES).

**Figure S5 subnetworks and GSEAs of the GSMs.**


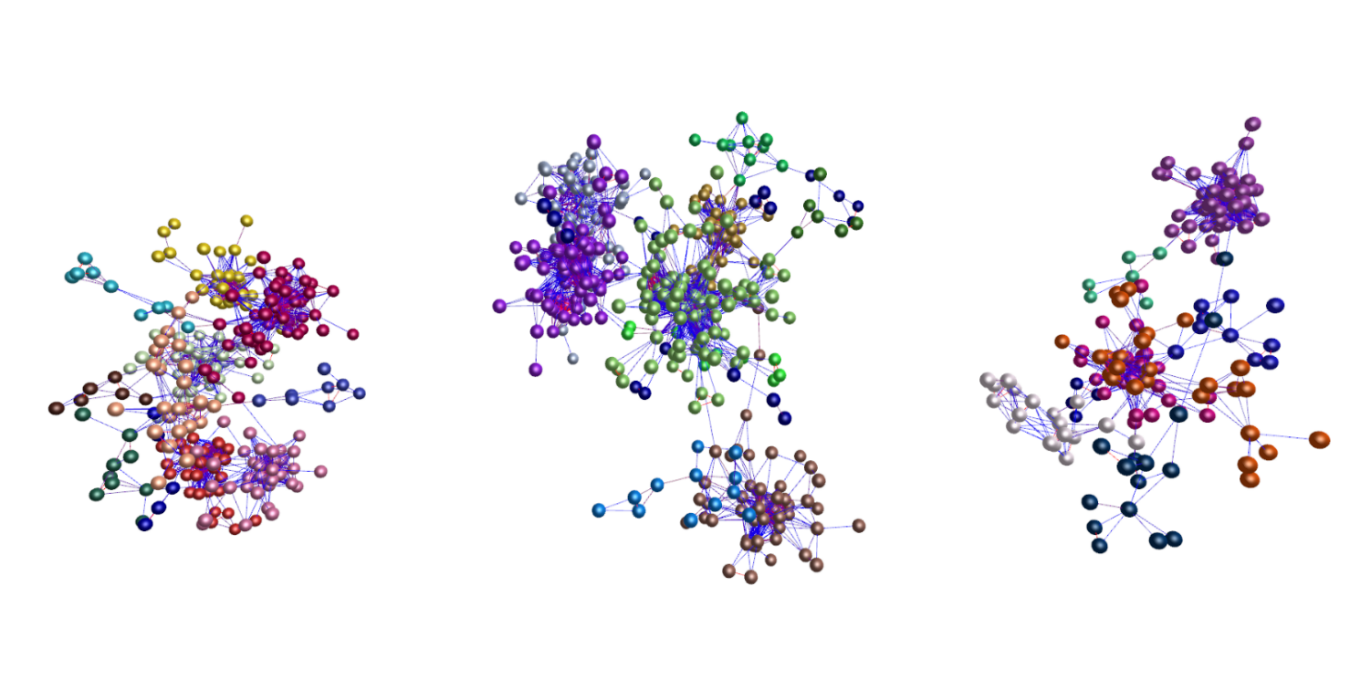

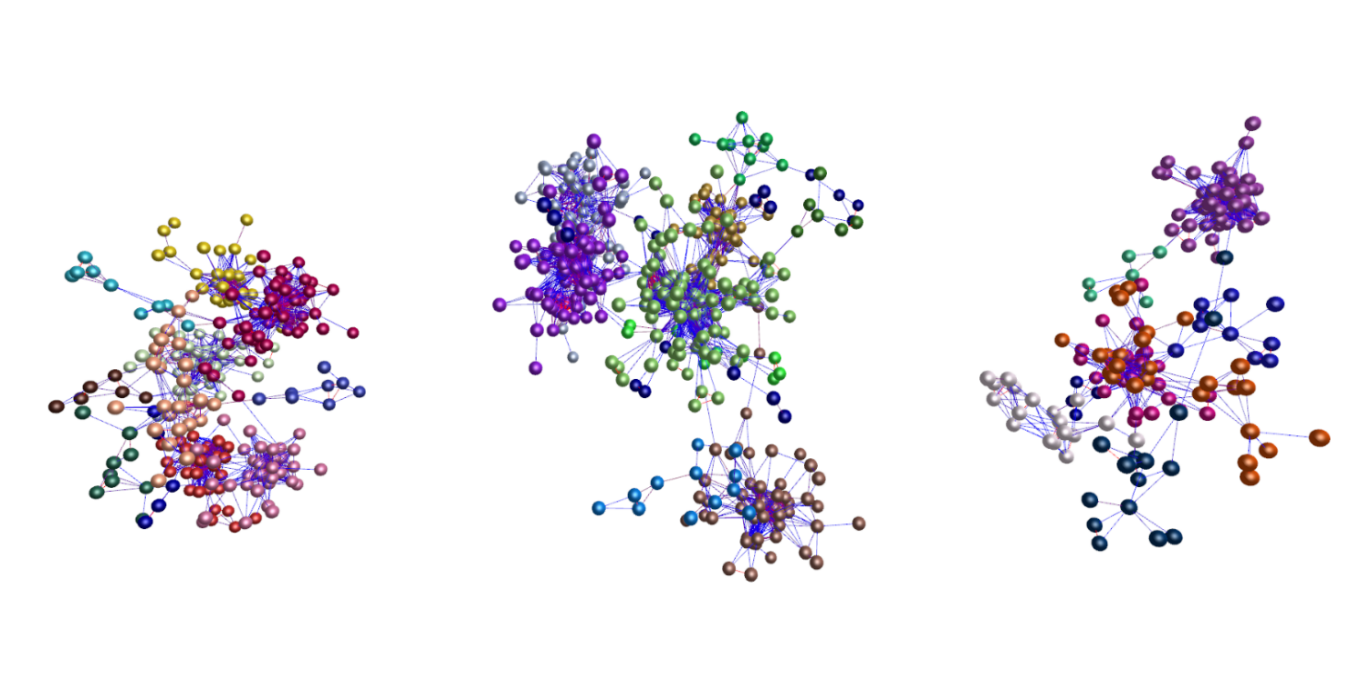


**6**

**12**

**13**

**9**

**10**


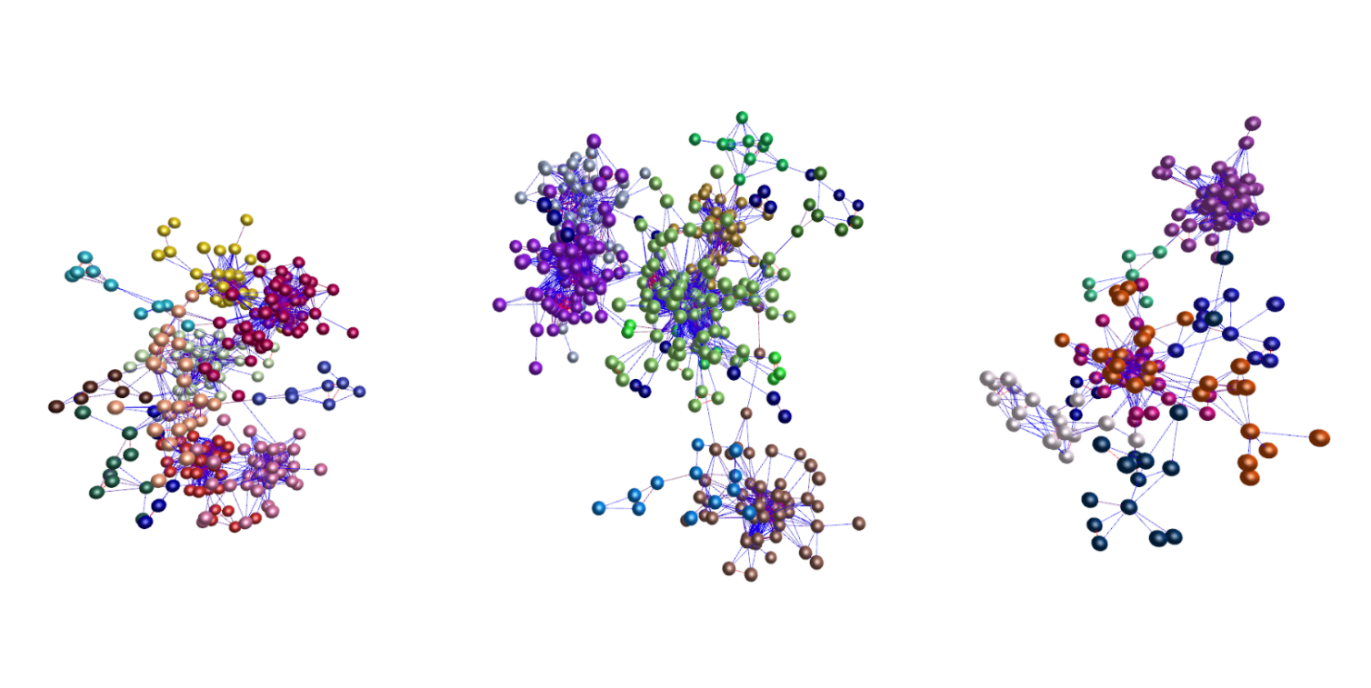


**4**

**1**

**2**

**3**

**7**

**5**

**11**

**8**

**A**

**B**

**C**


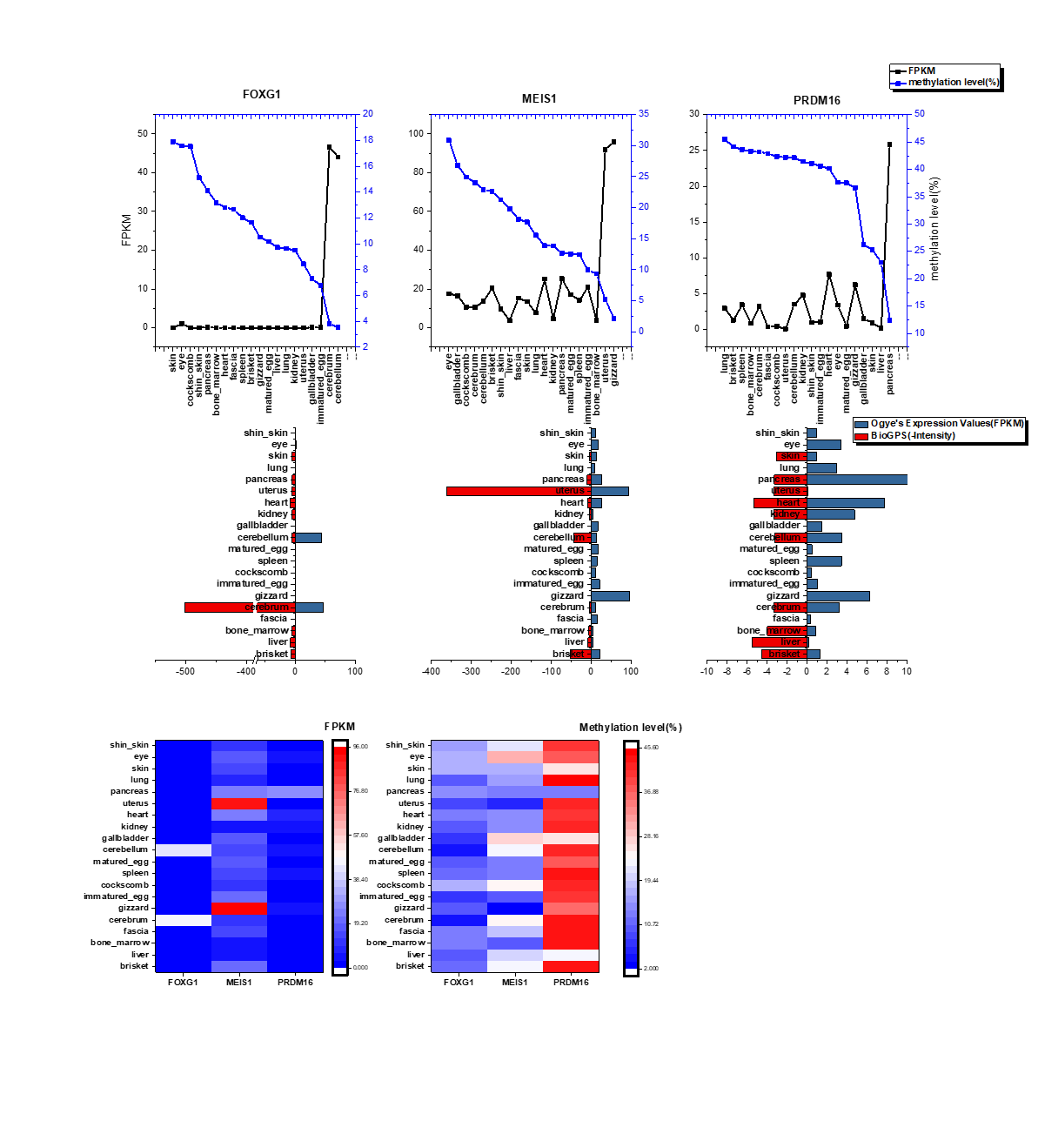


**C**

**B**

**Figure S6 Correlation and tissue specific characteristics of three genes which searching for GSMs in the imprintome database.**

(A) DNA methylation and Gene expression values per tissues. We sorted tissues based on DNA methylation levels. (B) Comparison of gene expression values of BioGPS and imprinted genes. The tissues that perform the same function in human were selected and compared with.( SmoothMuscle, Retina, Uterus, CerebellumPeduncles, Bonemarrow, Pancreas, SkeletalMuscle, Liver, Heart, Cerebellum, Skin and Kidney) (C) Comparison of DNA methylation and Gene expression values between the imprinted genes.


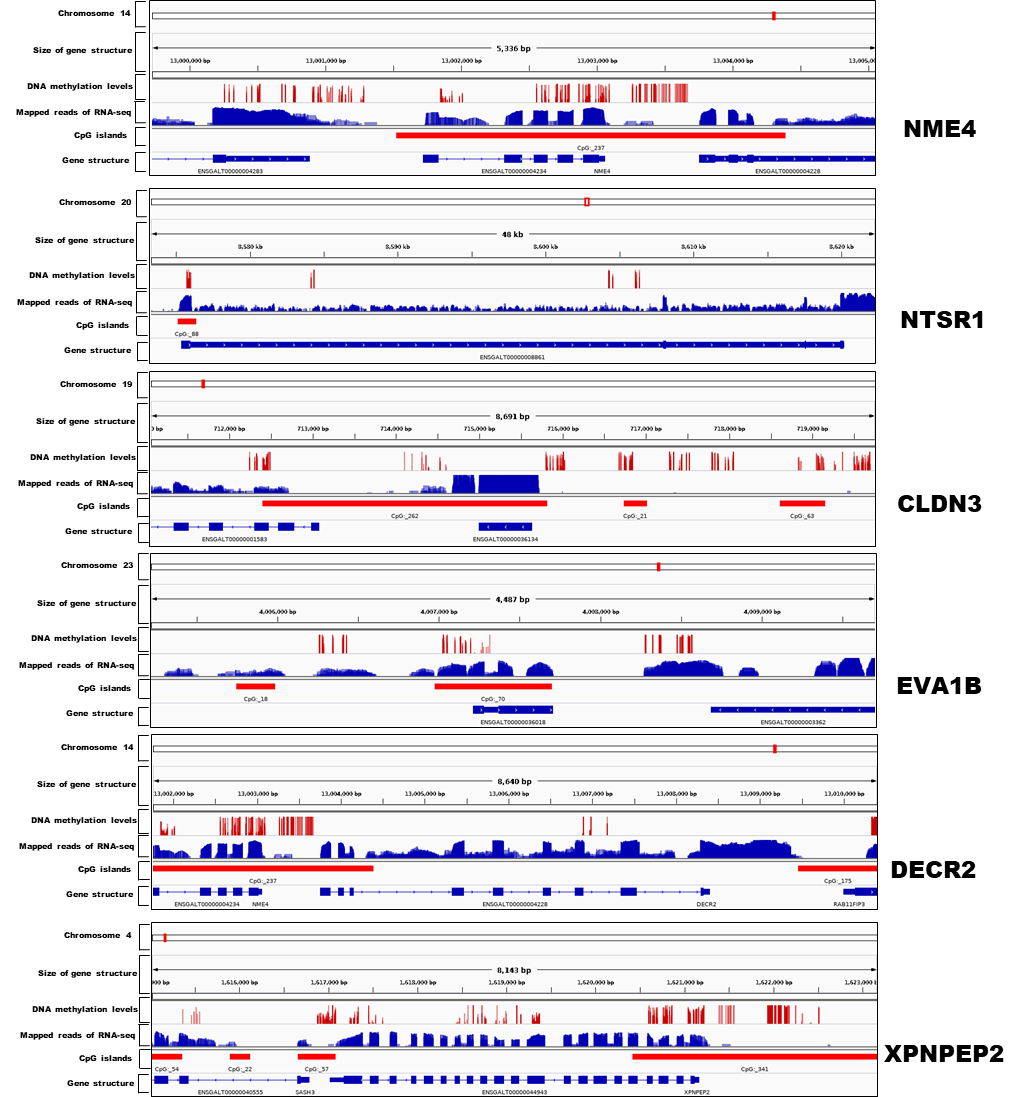


**Figure S7 Gene structures of the 6 highly correlated GSMs.**
